# Safety and Outcomes of Different Surgical Techniques for Cubital Tunnel Decompression: A Systematic Review and Network Meta-analysis

**DOI:** 10.1001/jamanetworkopen.2020.24352

**Published:** 2020-11-24

**Authors:** Ryckie G. Wade, Timothy T. Griffiths, Robert Flather, Nicholas E. Burr, Mario Teo, Grainne Bourke

**Affiliations:** 1 Department of Plastic and Reconstructive Surgery, Leeds Teaching Hospitals Trust, Leeds, United Kingdom; 2 Leeds Institute for Medical Research, University of Leeds, Leeds, United Kingdom; 3 Cancer Epidemiology Group, Institute of Cancer and Pathology and Institute of Data Analytics, University of Leeds, United Kingdom; 4 Hull University Teaching Hospitals NHS Trust, Hull, United Kingdom; 5 Bristol Institute of Clinical Neuroscience, Southmead Hospital, Bristol, United Kingdom

## Abstract

**Importance:**

Cubital tunnel syndrome is the second most common compressive neuropathy, affecting 6% of the population. Numerous different operations are performed globally to treat it; however, prior conventional (pairwise) meta-analyses have been unable to determine which procedure is associated with the best outcomes and fewest complications.

**Objective:**

To evaluate which operation for cubital tunnel syndrome is associated with the greatest likelihood of symptomatic cure.

**Data Sources:**

PubMed, EMBASE, and CENTRAL were searched from database inception to March 2, 2019, with no restrictions on the setting or design of studies.

**Study Selection:**

Experimental and observational studies directly comparing the outcomes of at least 2 surgical treatments for adults with primary cubital tunnel syndrome were included. Case reports were excluded, and when comparative studies had subgroups with 1 participant, the single-participant subgroup was excluded. The treatments had to be in situ decompression with or without medial epicondylectomy or an anterior subcutaneous, subfascial, intramuscular, or submuscular transposition. The access could be open, minimally invasive, or endoscopic. The comparator could be sham surgery or any operation mentioned earlier.

**Data Extraction and Synthesis:**

Data were extracted by 2 independent reviewers, following the Preferred Reporting Items for Systematic Reviews and Meta-analyses (PRISMA) reporting guideline and the PRISMA Network Meta-analysis extension statement. Network meta-analysis was used to estimate the relative efficacy and safety associated with interventions using relative risks. Surgical techniques were ranked by their probability of being the best (P score) and interpreted in terms of their clinical impact.

**Main Outcomes and Measures:**

The primary outcome was response to treatment (ie, symptomatic improvement). The secondary outcomes were perioperative complications, reoperation, and recurrence.

**Results:**

A total of 30 studies of 2894 limbs undergoing 8 different operations were included. Across the studies, 56% of participants were men, the mean (SD) age was 48 (8) years, and patients had symptoms for a mean (SD) of 15 (7) months. Overall, 87% (95% CI, 92%-91%) of patients improved with surgery; all forms of in situ decompression were more effective than any type of transposition procedure; for example, open in situ decompression with epicondylectomy was associated with higher success rates than subcutaneous transposition (relative risk, 1.13; 95% CI, 1.01-1.25). Postoperatively, 3% (95% CI, 2%-4%) of patients developed complications, and in situ decompressions were ranked as the least risky, although there was considerable uncertainty in this outcome. Overall, 2% (95% CI, 1%-3%) of patients required reoperation; open in situ decompression was associated with the fewest reoperations; in comparison, submuscular transposition was associated with 5 times the risk of reoperation (relative risk, 5.08; 95% CI, 2.06-12.52). During surveillance, 3% (95% CI, 1%-4%) of patients developed recurrence, and open in situ decompression with epicondylectomy was ranked as the safest operation, although there was uncertainty in the estimates.

**Conclusions and Relevance:**

In this network meta-analysis, open in situ decompression (with or without medial epicondylectomy) appeared to be the safest operation and also was associated with the best outcomes for patients with primary cubital tunnel syndrome. Future research should focus on better defining this disorder and developing core outcome measures.

## Key Points

**Question:**

For adults with primary cubital tunnel syndrome, which operation is associated with the best chance of symptomatic cure and lowest risk of complications?

**Findings:**

This network meta-analysis included 30 studies comparing 8 different operations in 2894 limbs. It found that 87% of patients improve with surgery and that open in situ decompression (with or without a medial epicondylectomy) was associated with the greatest response to treatment and lowest complication risk.

**Meaning:**

The findings of this study suggest that for adults with primary cubital tunnel syndrome, the most beneficial operation appears to be open in situ decompression.

## Teaser

This systematic review and network meta-analysis evaluates which operation for cubital tunnel syndrome is associated with the best chance of symptomatic cure and lowest risk of complications.

## Introduction

Cubital tunnel syndrome is the second most common compressive neuropathy, affecting up to 6% of the population^1^ or 36 per 100 000 person-years.^2^ Surgical decompression of the cubital tunnel is the most effective treatment.^3,4,5^ Consequently, approximately 15 000 people across the UK^6^ and US^7^ undergo surgical decompression annually.

There are numerous techniques for decompressing the ulnar nerve around the elbow, which include open, minimally invasive, and endoscopic approaches. Once the ulnar nerve is decompressed, to reduce traction on it in elbow flexion, resection of the medial epicondyle (epicondylectomy) may be performed, with or without anterior transposition of the ulnar nerve to a subcutaneous, subfascial, or submuscular position. Several factors inform surgeons’ choice of technique,^8^ and there are no clear indications for 1 approach over another. Therefore, most surgeons (86%) use more than 1 procedure in their treatment of patients with cubital tunnel syndrome.^9^ During the last decade, at least 15 systematic reviews and pairwise meta-analyses have failed to resolve uncertainty about the efficacy and safety of these different operations for primary cubital tunnel syndrome,^10,11,12,13,14,15,16,17,18,19,20,21,22^ which is manifested in persistent variation in practice.^23^ This uncertainty is important to resolve because as many as 30% of patients do not improve after surgery^24^ and many are subject to revision surgery, which is rarely curative.^25^

Network meta-analysis is a technique for comparing multiple treatments simultaneously by combining direct evidence from clinical studies and indirect evidence from within a network. This advanced form of meta-analysis has several distinct advantages over standard (pairwise) meta-analyses, including better precision and power,^26,27^ the ability to compare interventions that have not been directly compared before (ie, in a real-life head-to-head study), and the capacity to rank competing treatments to inform clinical decisions.^28^ Therefore, network meta-analysis has the potential to address some of the remaining uncertainties about the efficacy and safety associated with different operations for cubital tunnel syndrome. In this study, we aimed to rank the safety and outcomes of different techniques for adults with primary cubital tunnel decompression.

## Methods

This review was registered on the PROSPERO database (CRD42019127892); it was designed and conducted in accordance with the *Cochrane Handbook for Systematic Reviews of Interventions*, version 5.1.0^29^ and was written in accordance with the Preferred Reporting Items for Systematic Reviews and Meta-analyses (PRISMA) reporting guideline^30^ and the PRISMA Network Meta-analysis extension statement.^31^

### Study Selection

We included experimental and observational studies directly comparing the outcomes of at least 2 surgical treatments for adults (aged >16 years) with primary cubital tunnel syndrome. We excluded case reports, and when comparative studies had a subgroup with 1 participant, the single-participant subgroup was excluded. The intervention had to be 1 of the following open, minimally invasive, or endoscopic techniques: in situ decompression; in situ decompression with medial epicondylectomy; anterior subcutaneous, anterior subfascial, intramuscular, or submuscular transposition; or any combination thereof. The comparator could be sham surgery or any of the earlier mentioned techniques.

### Outcomes

The primary outcome was response to treatment. In the absence of a core outcome set, symptomatic improvement was measured with a variety of well-known tools, such as the McGowan, Bishop, Dellon, Yasutaka, and Wilson-Krout classifications. All tools assess similar parameters and broadly agree in cubital tunnel syndrome.^32^ They cannot be approximated to a scale, but changes after surgery (for better or worse) can be dichotomized into responders and nonresponders. We used the outcome measures in the original study to classify patients as responders or nonresponders. For example, if a patient’s McGowan score improved after surgery, they were classified as a responder. Conversely, if a patient’s McGowan score did not change or worsened after surgery, then they were defined as a nonresponder (treatment failure). When multiple outcome measures were reported, the patient-reported assessment was used because this is the most meaningful approach for patients.^33^ There was no minimum or maximum severity (clinical or electrodiagnostic) required for inclusion.

The secondary outcomes included short-term surgical site complications that warranted any form of medical or surgical intervention, including bleeding, infection, and wound dehiscence. Numbness around the surgical site was not considered a complication unless it was caused by the division of a named cutaneous nerve and treated by microsurgical neurorrhaphy. Reoperation was defined as repeated surgery for any reason (eg, evacuation of a hematoma, debridement of an infected or necrotic wound, revisional surgery for recurrence) and recurrence of symptoms (as defined by the original study) after a period of symptomatic relief, whether or not additional treatment was required.

### Search Strategy

PubMed, EMBASE, and CENTRAL were interrogated^34^ according to the search strategy in the eAppendix in the Supplement. No language restrictions were applied. Our searches yielded 1827 results in PubMed, 1508 in EMBASE, and 79 in CENTRAL on March 2, 2019. After deduplication, there were 522 citations, which were independently screened by 3 review authors (R.G.W., T.T.G., and R.F.). The full texts of all potentially relevant articles were obtained. The reference lists for included articles and previous systematic reviews^10,11,17,18,19,20,21,35^ were also reviewed. Included articles were compared and disagreements resolved by discussion.

### Nodes

Treatments were grouped into the following nodes: open in situ decompression, open in situ decompression and medial epicondylectomy, subcutaneous transposition, submuscular transposition, endoscopic in situ decompression, endoscopic subcutaneous transposition, intramuscular transposition, and speculum in situ decompression. One study^36^ reported a subfascial transposition, but the described surgical technique was identical to an anterior subcutaneous transposition and so data were assimilated in the subcutaneous transposition node.

### Data Extraction

Three review authors (R.G.W., T.T.G., and R.F.) extracted details of the study design, demographic characteristics, and statistics of interest. Where data were missing or unclear, the author(s) were contacted. The authors of 1 article^37^ provided data on request. In 1 study,^36^ 2 groups with single participants were discarded. In 1 study,^38^ we were unable to disaggregate the outcomes for intramuscular transposition and submuscular transposition, requiring the exclusion of these groups. The unit of analysis was the unit reported in the study; bilateral procedures are rarely performed simultaneously, so we considered that bilateral procedures (if not otherwise stated) were performed at times sufficiently separated to be considered independent.

### Risk of Bias Assessment

The risk of methodological bias was assessed by 3 authors (R.G.W., T.T.G., and R.F.) independently, using the Cochrane Risk of Bias tool^39^ (for randomized trials) or ROBINS-I tool^40^ (for observational studies). Assessments were displayed graphically with RevMan version 5 (Cochrane Collaboration) and the Confidence in Network Meta-Analysis^41^ tool. Disagreements were resolved by discussion.

### Assessing the Transitivity Assumption in Network Meta-analysis

An important concept of network meta-analysis is that all patients in a network should be equally eligible (in principle) to receive any of the treatments, a phenomenon that is typically termed jointly randomizable. This means that all patients in our networks should in principle be eligible to undergo any of the decompressive operations described. We assessed the validity of the transitivity assumption underlying the network meta-analysis conceptually by considering whether participants in the identified studies were jointly randomizable (ie, could in principle receive any of the treatments in the network) and whether the distribution of effect modifiers was similar across nodes.^27^ In this case, an effect modifier is a factor that changes the effectiveness of surgery. For example, it is noted that older patients benefit less from decompressive surgery than younger patients; therefore, age might modify the effectiveness of surgery. We tested the distribution of commonly espoused effect modifiers (ie, age, sex, and symptom severity) to ensure that they were balanced and thus our estimates were not confounded.

### Statistical Analysis

We produced a network plot to summarize the treatments followed by a series of frequentist, random-effects, network meta-analyses, using the netmeta package in R version 3 (R Project for Statistical Computing)^42^ and assuming a single heterogeneity parameter. To assess the agreement between randomized and nonrandomized evidence, we first performed separate network meta-analyses and compared the results.^43^ Because no important discrepancies were observed, we performed a joint analysis that included both study types (so-called naive network meta-analysis). Interventions were ranked by their P scores^44^ with the netrank function; P scores are assumed to take a value between 0 and 1, with a higher score indicating a better treatment.^44^ With the netleague package, network meta-analysis results are summarized in league tables and treatments ordered by their P score. Forest plots of relative risks and 95% CIs were generated with open in situ decompression as the reference treatment. Heterogeneity was quantified through the standard deviation of random effects (τ, assumed common for all comparisons in the network). Inconsistency was assessed with both global and local methods with the netsplit package^45,46^ and displayed via heat plots^45,47^ with the netheat command. We produced forest plots to show the relative risk and 95% CIs for the outcomes of interest, with open in situ decompression as the reference operation. To assess possible small study effects for the primary outcome, we produced a comparison adjusted funnel plot^48^ in Stata version 15 (StataCorp)^48^ with the netfunnel package.

Next, we performed a series of designed-adjusted analyses,^43^ whereby data from randomized studies were combined with data from nonrandomized studies after down-weighting of the effect of the latter. These analyses involved a variance-inflation factor^43^ (ie, an extra parameter used to increase the variance of nonrandomized studies), thus reducing their effect on the pooled network meta-analysis estimate. We used the following variance inflation factors: *w* *=* 1 (corresponding to the naive network meta-analysis [ie, including all studies at face value]), 0.8, 0.6, 0.4, 0.2, and 0 [ie, 0 excluded nonrandomized studies from the analysis]). Randomized clinical trials were not down-weighted in these designed-adjusted analyses. We produced forest plots with the results of all treatments vs the reference (open in situ decompression) for all analyses to show how gradually allowing nonrandomized evidence to inform the estimates of relative treatment effects. In our designed-adjusted analyses, we did not adjust the point estimates from nonrandomized studies^43^ because we could not be confident of the direction and magnitude of potential bias in the treatment effects.

Given that the secondary outcomes were rare, we used sensitivity fixed-effects Mantel-Haenszel network meta-analyses^49^ (using the netmetabin package), which synthesize odds ratios; however, for rare events, odds and risks are almost identical. Inconsistency in these networks was assessed with the netsplit package and SIDDE approach.^49^ The RStudio version 1.3 (R Project for Statistical Computing) package metaprop^50^ was used to estimate the pooled prevalence of outcomes, using Hartung-Knapp-Sidik-Jonkman random effects and the Freeman-Tukey double arcsine transformation to stabilize the variances of proportions close to 0 or 1.

Recent publications have highlighted problems with null hypothesis testing,^51,52^ particularly in network meta-analysis.^53^ Therefore, we did not use the concept of statistical significance when presenting or discussing results from network meta-analyses but instead focused on the clinical interpretation in relation to the corresponding point estimates and their respective confidence intervals.

## Results

### Study Selection

After review of 68 full texts, 38 articles were excluded with reasons (eFigure 1 in the Supplement), and 30 studies^36,37,38,54,55,56,57,58,59,60,61,62,63,64,65,66,67,68,69,70,71,72,73,74,75,76,77,78,79,80^ describing 8 operations were included.

### Study Characteristics

eTable 1 in the Supplement shows that there were 2894 limbs (belonging to ≥ 2675 patients) derived from 6 randomized trials,^56,57,59,64,68,77^ 1 quasi-randomized clinical trial,^78^ 3 prospective cohort studies,^60,62,71^ 14 retrospective cohort studies,^36,37,38,54,55,58,61,63,66,67,70,72,74,75^ and 6 studies that did not describe the design.^65,69,73,76,79,80^ Across the included studies, 56% were men, the mean (SD) age was 48 (8) years, and patients had symptoms for a mean (SD) of 15 (7) months.

### Risk of Bias Within Studies

The average risk of bias contributions for each comparison within the network are shown in eFigure 2 in the Supplement. The assessments of the risk of methodological bias for randomized clinical trials and nonrandomized studies are shown in eFigure 3 and eFigure 4 in the Supplement, respectively.

### Assessment of Transitivity

After grouping the studies by treatment comparison and inspecting the distribution of possible effect modifiers, there were no significant differences between the demographic characteristics or preoperative McGowan grades for all treatments (eTable 2 in the Supplement). Therefore, they were judged to be sufficiently similar to be jointly synthesized in a network meta-analysis.

### Agreement Between Randomized and Nonrandomized Studies

eFigure 5 and eFigure 6 in the Supplement show how the estimates derived from a network meta-analysis of only randomized controlled trials compare with a network meta-analysis of nonrandomized studies. The graphs showed no discrepancies between randomized and nonrandomized evidence. This was further corroborated after testing for differences between the 2 estimates for each treatment comparison (*P* > .05 for all χ^2^ tests). Thus, there was no evidence of incompatibility between randomized and nonrandomized evidence, so we proceeded with a joint (ie, naive) analysis. The randomized and nonrandomized studies contributing to the analyses are disaggregated in eFigure 7 and eFigure 8 in the Supplement.

### Response to Treatment

The network was composed of 30 studies,^36,37,38,54,55,56,57,58,59,60,61,62,63,64,65,66,67,68,69,70,71,72,73,74,75,76,77,78,79,80^ with 37 direct comparisons of 8 surgical techniques (Figure). Subcutaneous transposition was the most common operation (n = 1101 [38%]), followed by open in situ decompression (n = 803 [28%]), submuscular transposition (n = 397 [14%]), and endoscopic in situ decompression (n = 361 [12%]), with the remaining limbs treated by other techniques. Overall, 87% of patients improved with surgery (95% CI, 82%-91%; *I*^2^, 85%), and in situ decompressions (whether performed by an open, endoscopic, or minimally invasive approach) were superior to any type of transposition. Specifically, open in situ decompression and medial epicondylectomy was ranked as the best technique (P score, 0.8787), with a 13% (95% CI, 1%-25%) higher chance of cure than with subcutaneous transposition. Detailed results for all treatment comparisons are shown in Table 1. The estimated heterogeneity of the network was small (τ^2^ = 0.003); however, the local (ie, back-calculation) method identified inconsistency between the direct and indirect evidence for open in situ decompression and subcutaneous transposition (eFigure 9 and eTable 3 in the Supplement).

**Figure.**
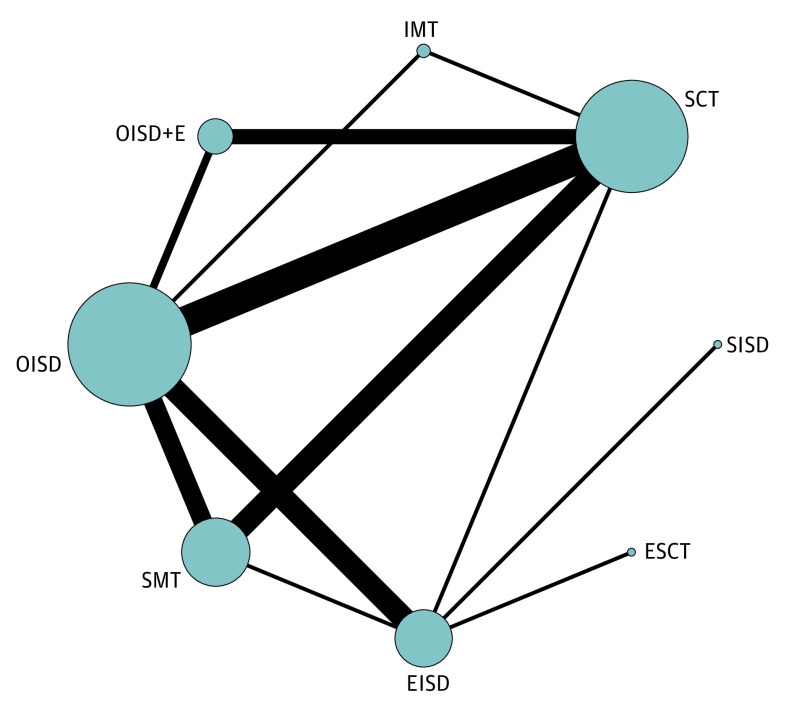
Studies Reporting Response to Treatment The size of the nodes corresponds to the number of patients. The thickness of the connecting lines corresponds to the number of studies. Techniques included open in situ decompression (OISD; 6 randomized clinical trials, 11 nonrandomized studies, 633 patients), subcutaneous transposition (SCT; 2 randomized clinical trials, 13 nonrandomized studies, 933 patients), submuscular transposition (SMT; 2 randomized clinical trials, 8 nonrandomized studies, 321 patients), endoscopic in situ decompression (EISD; 3 randomized clinical trials, 5 nonrandomized studies, 308 patients), open in situ decompression with medial epicondylectomy (OISD+E; 1 randomized clinical trial, 3 nonrandomized studies, 100 patients), endoscopic subcutaneous transposition (ESCT; 1 nonrandomized study, 52 patients), intramuscular transposition (IMT; 1 nonrandomized study, 9 patients), and speculum in situ decompression (SISD; 1 nonrandomized study, 15 patients).

**Table 1.** League Table of Pairwise Comparisons in Network Meta-analysis for the Relative Risk (With 95% CIs) of Responding to Treatment (ie, Improving)^a^

Open in situ decompression with epicondylectomy (P score, 0.8787)		0.91 (0.76-1.09)				1.21 (1.08-1.36)	
1.07 (0.81-1.42)	Speculum in situ decompression (P score, 0.5784)		1.03 (0.80-1.32)				
1.09 (0.98-1.22)	1.02 (0.78-1.32)	Open in situ decompression (P score, 0.5777)	1.01 (0.94-1.08)	1.03 (0.76-1.40)	1.01 (0.94-1.09)	1.04 (0.98-1.10)	
1.10 (0.97-1.25)	1.03 (0.80-1.32)	1.01 (0.94-1.07)	Endoscopic in situ decompression (P score, 0.5110)		0.97 (0.85-1.09)	1.02 (0.90-1.16)	1.11 (0.84-1.47)
1.10 (0.83-1.47)	1.03 (0.71-1.50)	1.01 (0.77-1.32)	1.01 (0.76-1.32)	Intramuscular transposition (P score, 0.4521)		1.03 (0.78-1.36)	
1.11 (0.99-1.26)	1.04 (0.80-1.36)	1.02 (0.96-1.09)	1.01 (0.93-1.10)	1.01 (0.77-1.32)	Submuscular transposition (P score, 0.4119)	1.00 (0.93-1.09)	
1.13 (1.01-1.25)	1.05 (0.81-1.37)	1.03 (0.81-1.09)	1.03 (0.95-1.11)	1.02 (0.78-1.33)	1.01 (0.95-1.08)	Subcutaneous transposition (P score, 0.3203)	
1.22 (0.90-1.66)	1.14 (0.78-1.66)	1.12 (0.84-1.49)	1.11 (0.84-1.47)	1.11 (0.75-1.63)	1.01 (0.75-1.46)	1.08 (0.81-1.44)	Endoscopic subcutaneous transposition (P score, 0.2351)

^a^ Treatments are ranked by their chance (P score) of improving symptoms; the top left is the best, whereas the bottom right is the worst. Estimates on the upper right are direct comparisons (ie, head-to-head studies); the lower-left estimates are from the network meta-analysis. A relative risk greater than 1 means that the risk of the event was higher in the row-defining treatment.

### Surgical Site Complications

The network was composed of 25 studies^36,55,56,57,58,59,60,61,62,63,65,67,69,70,71,72,73,74,75,77,78,79^ with 22 direct comparisons of complications after 6 different operations (eFigure 10 in the Supplement). Overall, 3% of patients developed a postoperative complication (95% CI, 2%-4%; *I*^2^, 55%). Endoscopic in situ decompression was ranked as the most hazardous operation (ie, most likely to result in complications), whereas open in situ decompression and medial epicondylectomy was the least. Detailed results are shown in Table 2. There was no measurable heterogeneity (τ^2^ = 0) (eTable 4 in the Supplement) or inconsistency within the network (eFigure 11 in the Supplement).

**Table 2.** League Table of Pairwise Comparisons in Network Meta-analysis for the Relative Risk (With 95% CIs) of Complications^a^

Open in situ decompression with epicondylectomy (P score, 0.1494)				5.81 (0.72-46.90)			
8.10 (0.77-85.46)	Intramuscular transposition (P score, 0.3033)			2.51 (0.11-57.42)		4.70 (0.24-90.29)	
7.10 (0.75-67.12)	4.61 (0.21-100.19)	Open in situ decompression (P score, 0.3582)		1.68 (0.89-3.16)		1.69 (0.65-4.42)	2.25 (0.90-5.64)
8.57 (0.41-179.33)	4.04 (0.22-72.75)	2.25 (0.90-5.64)	Speculum in situ decompression (P score, 0.3857)				3.29 (0.14-76.39)
5.81 (0.72-46.90)	4.87 (0.13-183.88)	1.97 (0.90-4.33)	3.29 (0.14-76.39)	Subcutaneous transposition (P score, 0.6193)		1.26 (0.42-3.81)	
2.46 (0.05-125.12)	3.30 (0.18-61.59)	2.38 (0.28-20.03)	2.89 (0.10-83.82)	1.39 (0.47-4.15)	Endoscopic subcutaneous transposition (P score, 0.7109)		0.95 (0.14-6.47)
3.61 (0.41-31.55)	1.40 (0.02-114.08)	1.61 (0.90-2.90)	3.48 (0.09-138.85)	1.22 (0.53-2.80)	0.95 (0.14-6.47)	Submuscular transposition (P score, 0.7147)	
1.76 (0.05-64.00)	2.05 (0.11-38.75)	0.68 (0.03-18.06)	2.36 (0.08-65.83)	1.47 (0.16-13.45)	0.83 (0.09-8.04)	1.14 (0.34-3.83)	Endoscopic in situ decompression (P score, 0.7585)

^a^ Treatments are ranked by their chance (P score) of causing complications; the top left is the best, whereas the bottom right is the worst. Estimates on the upper right are direct comparisons (ie, head-to-head studies); the lower-left estimates are from the network meta-analysis. A relative risk greater than 1 means that the risk of the event was higher in the row-defining treatment.

A sensitivity fixed-effects Mantel-Haenszel network meta-analysis yielded similar findings (eTable 5 in the Supplement) and again showed that open in situ decompression was associated with fewer complications than transposition and endoscopic or minimally invasive procedures. There was still no measurable heterogeneity (τ^2^ = 0) (eTable 6 in the Supplement) or inconsistency within the network (eFigure 12 in the Supplement).

### Reoperation

Reoperation was reported in 17 studies^38,55,56,61,62,63,64,65,66,68,70,71,72,75,77,78,79^; however, because of the rate of zero-event groups and the overall rarity of reoperation, only 7 studies^38,55,68,72,75,77,78^ could be synthesized in a fixed-effects Mantel-Haenszel network meta-analysis of 5 different treatments, with 15 direct comparisons (eFigure 13 in the Supplement). During follow-up, 2% of patients required revision surgery (95% CI, 1%-3%; *I*^2^, 61%). With 95% probability, submuscular transposition was the most hazardous technique, with 5 times the risk of reoperation compared with open in situ decompression (relative risk, 5.08; 95% CI, 2.06-12.52). Detailed comparisons of treatments are provided in Table 3. There was no measurable heterogeneity (τ^2^ = 0) (eTable 7 in the Supplement) or inconsistency within the network (eFigure 14 in the Supplement).

**Table 3.** League Table of Pairwise Comparisons in Network Meta-analysis for the Relative Risk (With 95% CIs) of Reoperation at the Same Surgical Site for Any Reason^a^

Open in situ decompression (P score, 0.2168)		1.59 (0.49-5.11)	0.55 (0.21-1.48)	0.20 (0.07-0.54)
0.83 (0.06-12.25)	Endoscopic subcutaneous transposition (P score, 0.2834)	1.92 (0.17-21.88)		
1.59 (0.49-5.11)	0.52 (0.05-5.91)	Endoscopic in situ decompression (P score, 0.4887)		
1.89 (0.73-4.87)	0.44 (0.03-7.61)	0.84 (0.19-3.78)	Subcutaneous transposition (P score, 0.5643)	0.31 (0.06-1.55)
5.08 (2.06-12.52)	0.16 (0.01-2.79)	0.31 (0.07-1.37)	0.37 (0.12-1.18)	Submuscular transposition (P score, 0.9468)

^a^ Treatments are ranked by their chance (P score) of need for reoperation; the top left is the best, whereas the bottom right is the worst. Estimates on the upper right are direct comparisons (ie, head-to-head studies); the lower-left estimates are from the network meta-analysis. A relative risk greater than 1 means that the risk of the event was higher in the row-defining treatment.

### Recurrence

Overall, 15 studies^36,38,55,56,63,64,65,66,67,68,72,75,77,78,79^ reported recurrence, providing 19 direct comparisons of 8 operations (eFigure 15 in the Supplement). During surveillance, 3% of patients developed recurrent symptoms (95% CI, 1%-4%; *I*^2^, 66%). Open in situ decompression and medial epicondylectomy was ranked as the best technique with the lowest risk of recurrence. Conversely, and with 78% probability, submuscular transposition was the most hazardous operation and was associated with the highest risk of recurrence (Table 4). There was no measurable heterogeneity (τ^2^ = 0.93) (eTable 8 in the Supplement) or inconsistency within the network (eFigure 16 in the Supplement).

**Table 4.** League Table of Pairwise Comparisons in Network Meta-analysis for the Relative Risk (With 95% CIs) of Recurrent Cubital Tunnel Syndrome^a^

Open in situ decompression with epicondylectomy (P score, 0.2157)		3.00 (0.09-100)	3.53 (0.10-129)	6.74 (0.21-213)			
2.73 (0.03-297)	Intramuscular transposition (P score, 0.4377)		2.51 (0.07-88.6)				2.82 (0.08-98.9)
3.00 (0.09-100)	1.10 (0.00-384)	Subfascial transposition (P score, 0.4407)					
4.75 (0.17-133)	1.74 (0.06-49.6)	1.58 (0.01-201)	Subcutaneous transposition (P score, 0.4599)	0.97 (0.30-3.10)			3.44 (0.70-16.8)
5.42 (0.20-147)	1.98 (0.06-61.8)	1.81 (0.01-224)	1.14 (0.38-3.43)	Open in situ decompression (P score, 0.5115)	1.05 (0.17-6.59)		2.01 (0.40-10.1)
5.71 (0.13-250)	2.09 (0.04-103)	1.90 (0.01-331)	1.20 (0.14-10.2)	1.05 (0.17-6.59)	Endoscopic in situ decompression (P score, 0.5442)	1.06 (0.04-26.77)	
6.04 (0.04-871)	2.21 (0.01-349)	2.01 (0.00-886)	1.27 (0.03-61.2)	1.11 (0.03-45.8)	1.06 (0.04-26.8)	Endoscopic subcutaneous transposition (P score, 0.5982)	
11.10 (0.34-363)	4.06 (0.14-115)	3.70 (0.03-522)	2.33 (0.62-8.76)	2.05 (0.54-7.74)	1.94 (0.20-18.7)	1.84 (0.04-95.1)	Submuscular transposition (P score, 0.7920)

^a^ Treatments are ranked by their chance (P score) of recurrence; the top left is the best, whereas the bottom right is the worst. Estimates on the upper right are direct comparisons (ie, head-to-head studies); the lower-left estimates are from the network meta-analysis. A relative risk greater than 1 means that the risk of the event was higher in the row-defining treatment.

A sensitivity fixed-effects Mantel-Haenszel network meta-analysis yielded similar findings (eTable 9 in the Supplement). There was still no measurable heterogeneity (τ^2^ = 0) (eTable 10 in the Supplement) or inconsistency within the network (eFigure 17 in the Supplement).

### Small-Study Effects

An adjusted funnel plot showed no evidence of small-study effects. eFigure 18 in the Supplement presents the details.

### Assessing Confidence in Results From the Analyses

There was moderate confidence in the mixed evidence but low confidence in the indirect evidence. eTable 11 in the Supplement presents the details.

## Discussion

This systematic review and network meta-analysis found that open in situ decompression with or without medial epicondylectomy was associated with the greatest response to treatment and the lowest risk of complications, reoperation, and recurrence. Our network meta-analysis provides a central reference point for the global evidence on cubital tunnel syndrome surgery to help inform clinician practice, training, and international guidelines.

Our findings show that in situ decompression (whether by open, endoscopic, or minimally invasive means) was associated with lower risk of complications than any form of transposition procedure for primary cubital tunnel syndrome (Table 2); furthermore, the addition of an epicondylectomy was associated with an increased probability of symptomatic cure without increasing the risks of complications. The 95% CIs around these estimates are narrow, indicating a high degree of certainty, which is corroborated by the sensitivity analysis. Clearly, selecting an operation with the highest success rate and lowest complication risk is beneficial to patients. The reduced operative time and complexity of in situ decompression^77,81^ are also beneficial to surgeons. Furthermore, health care services stand to gain from our findings because in situ decompressions are 18% to 55% less expensive than transposition procedures,^81^ a relative cost saving that does not include the direct and indirect savings that come from avoiding complications, reoperation, and recurrence, which are more common in transposition surgeries. Whether the addition of an epicondylectomy to an in situ decompression increases the direct cost is unclear and needs exploring. However, it is plausible that any increase in surgical time and cost may be offset by a lower risk of complications and reoperation. Overall, the results suggest that in situ decompression (with or without a medial epicondylectomy) is the most effective and safe operation for primary cubital tunnel syndrome.

This review has identified important deficiencies in the literature. First, stakeholders must reach consensus on the definition of cubital tunnel syndrome, with or without classification-system-based patient-reported outcomes measures that have constructive validity. Second, a set of core outcome measures is needed to complement work on the minimal clinical important differences in ulnar neuropathy.^82^ Thereafter, we echo calls^13,83^ for comparative studies of operative vs nonoperative treatments. There is a paucity of data on nonoperative management,^84^ and we have a responsibility to inform patients about the evidence for and against all management options.

### Limitations

This study has limitations. The surveillance period used in most studies is arguably insufficient to capture all cases of reoperation and recurrence because relapse typically occurs between 6 and 21 months postoperatively.^25^ Therefore, our estimates may underestimate the true prevalence of recurrence, which, compounded by biases of attrition and reporting, may misrepresent the true risk of recurrence for a given procedure. As such, we recommend cautious interpretation of these outcomes.

Ideally, the analyses of response to treatment would have included nonoperative treatments, although this might violate transitivity assumptions, given that some surgeons may not accept or offer nonoperative treatment to patients with moderate or severe cubital tunnel syndrome.

Bilateral surgery was described in 6 studies,^57,66,68,70,74,75^ which raises concerns about the unit of analysis^85^ and makes it impossible to judge how this may have affected our network meta-analyses. Despite this, it is likely that bilateral operations were performed at times sufficiently separated to be considered independent events, and all studies that reported bilateral operations used the same procedure on both limbs.

We transformed binomial data (to pool) with the Freeman-Tukey method because it stabilizes the variances of proportions close to 0 or 1; however, this method can yield unreliable estimates when back-transformed. Similarly, we used the DerSimonian-Laird method to synthesize binomial data, and this can induce biased estimates with falsely high precision; better methods exist but are not yet available. Therefore, caution is recommended when the pooled prevalence of outcomes is interpreted.

## Conclusions

Overall, the results of this study suggest that the rate of cure for patients with cubital tunnel syndrome who receive surgery is high and complications are uncommon. According to the available evidence and notwithstanding some uncertainty regarding the estimates, open in situ decompression (with or without medial epicondylectomy) appeared to be the best procedure for patients with primary cubital tunnel syndrome. We suggest that future research focus on defining the disorder and generating core outcome measures before further (necessary) comparative studies are undertaken.

## Notes

Supplement. **eFigure 1.** Study Selection Flowchart **eFigure 2.** The Average Risk of Bias Contributions for Each Comparison **eFigure 3. **Risk of Bias Summary for Randomized Studies **eFigure 4.** Risk of Bias Summary for Nonrandomized Studies **eFigure 5.** Design-Adjusted Analyses for Response to Treatment **eFigure 6.** Forest Plots for Response to Treatment **eFigure 7.** Network Plot of Randomized Studies Included in Analysis for Response to Treatment **eFigure 8.** Network Plot of Nonrandomized Studies Included in Analysis for Response to Treatment **eFigure 9.** Network Heat Plot for Response to Treatment **eFigure 10.** Network Plot of Studies Included in the Analysis of Complications **eFigure 11.** Network Heat Plot for Complications (Naive Random-Effects NMA) **eFigure 12.** Network Heat Plot for Complications (Naive Fixed-Effects NMA) **eFigure 13.** Network Plot of Studies Included in the Analysis of Reoperation **eFigure 14. **Network Heat Plot for Reoperation (Naive Fixed-Effects Mantel-Haenszel NMA) **eFigure 15.** Network Plot of Studies Included in the Analysis of Recurrence **eFigure 16. **Network Heat Plot for Recurrence (Naive Random-Effects NMA) **eFigure 17. **Network Heat Plot for Recurrence (Naive Fixed-Effects Mantel-Haenzel NMA) **eFigure 18.** Comparison-Adjusted Funnel Plot **eTable 1.** Summary of Study Characteristics **eTable 2.** Summary of Variables That Might Moderate the Relative Effects of Treatments **eTable 3.** Direct and Indirect Estimates From the Random-Effects NMA of Response to Treatment **eTable 4.** Direct and Indirect Estimates From the Random-Effects NMA of Complications **eTable 5.** League Table of Pairwise Comparisons for Complications (Fixed-Effects Mantel-Haenszel) **eTable 6.** Direct and Indirect Estimates From the Fixed-Effects Mantel-Haenszel NMA of Complications **eTable 7.** Comparisons of the Direct and Indirect Estimates From the Fixed-Effects NMA of Reoperation **eTable 8.** Direct and Indirect Estimates From the Random-Effects NMA of Recurrence **eTable 9.** League Table of Pairwise Comparisons for Recurrence (Fixed-Effects Mantel-Haenszel) **eTable 10.** Direct and Indirect Estimates From the Fixed-Effects Mantel-Haenszel NMA of Recurrence **eTable 11.** CINEMA Assessments for the Primary Outcome **eAppendix. **Search Results
