# Supplementary material for: Safety and Outcomes of Different Surgical Techniques for Cubital Tunnel Decompression: A Systematic Review and Network Meta-analysis
Source: JAMA Netw Open. 2020 Nov 24;3(11):e2024352. doi: 10.1001/jamanetworkopen.2020.24352 (PMC7686867; doi:10.1001/jamanetworkopen.2020.24352)
Supplement: Supplement. — eFigure 1. Study Selection Flowchart eFigure 2. The Average Risk of Bias Contributions for Each Comparison eFigure 3. Risk of Bias Summary for Randomized Studies eFigure 4. Risk of Bias Summary for Nonrandomized Studies eFigure 5. Design-Adjusted Analyses for Response to Treatment eFigure 6. Forest Plots for Response to Treatment eFigure 7. Network Plot of Randomized Studies Included in Analysis for Response to Treatment eFigure 8. Network Plot of Nonrandomized Studies Included in Analysis for Response to Treatment eFigure 9. Network Heat Plot for Response to Treatment eFigure 10. Network Plot of Studies Included in the Analysis of Complications eFigure 11. Network Heat Plot for Complications (Naive Random-Effects NMA) eFigure 12. Network Heat Plot for Complications (Naive Fixed-Effects NMA) eFigure 13. Network Plot of Studies Included in the Analysis of Reoperation eFigure 14. Network Heat Plot for Reoperation (Naive Fixed-Effects Mantel-Haenszel NMA) eFigure 15. Network Plot of Studies Included in the Analysis of Recurrence eFigure 16. Network Heat Plot for Recurrence (Naive Random-Effects NMA) eFigure 17. Network Heat Plot for Recurrence (Naive Fixed-Effects Mantel-Haenzel NMA) eFigure 18. Comparison-Adjusted Funnel Plot eTable 1. Summary of Study Characteristics eTable 2. Summary of Variables That Might Moderate the Relative Effects of Treatments eTable 3. Direct and Indirect Estimates From the Random-Effects NMA of Response to Treatment eTable 4. Direct and Indirect Estimates From the Random-Effects NMA of Complications eTable 5. League Table of Pairwise Comparisons for Complications (Fixed-Effects Mantel-Haenszel) eTable 6. Direct and Indirect Estimates From the Fixed-Effects Mantel-Haenszel NMA of Complications eTable 7. Comparisons of the Direct and Indirect Estimates From the Fixed-Effects NMA of Reoperation eTable 8. Direct and Indirect Estimates From the Random-Effects NMA of Recurrence eTable 9. League Table of Pairwise Comparisons for Recurrence (Fixed- [file jamanetwopen-e2024352-s001.pdf]

Supplemental Online Content

Wade RG, Griffiths TT, Flather R, Burr NE, Teo M, Bourke G. Safety and outcomes of different surgical techniques for cubital tunnel decompression: a systematic review and network meta-analysis. *JAMA Network Open*. 2020;3(11):e2024352.

doi:10.1001/jamanetworkopen.2020.24352

eFigure 1. Study Selection Flowchart

eFigure 2. The Average Risk of Bias Contributions for Each Comparison

eFigure 3. Risk of Bias Summary for Randomized Studies

eFigure 4. Risk of Bias Summary for Nonrandomized Studies

eFigure 5. Design-Adjusted Analyses for Response to Treatment

eFigure 6. Forest Plots for Response to Treatment

eFigure 7. Network Plot of Randomized Studies Included in Analysis for Response to Treatment

eFigure 8. Network Plot of Nonrandomized Studies Included in Analysis for Response to Treatment

eFigure 9. Network Heat Plot for Response to Treatment

eFigure 10. Network Plot of Studies Included in the Analysis of Complications

eFigure 11. Network Heat Plot for Complications (Naive Random-Effects NMA)

eFigure 12. Network Heat Plot for Complications (Naive Fixed-Effects NMA)

eFigure 13. Network Plot of Studies Included in the Analysis of Reoperation

eFigure 14. Network Heat Plot for Reoperation (Naive Fixed-Effects Mantel-Haenszel NMA)

eFigure 15. Network Plot of Studies Included in the Analysis of Recurrence

eFigure 16. Network Heat Plot for Recurrence (Naive Random-Effects NMA)

eFigure 17. Network Heat Plot for Recurrence (Naive Fixed-Effects Mantel-Haenzel NMA)

eFigure 18. Comparison-Adjusted Funnel Plot

eTable 1. Summary of Study Characteristics

eTable 2. Summary of Variables That Might Moderate the Relative Effects of Treatments

eTable 3. Direct and Indirect Estimates From the Random-Effects NMA of Response to Treatment

eTable 4. Direct and Indirect Estimates From the Random-Effects NMA of Complications

eTable 5. League Table of Pairwise Comparisons for Complications (Fixed-Effects Mantel-Haenszel)

© 2020 Wade RG et al. *JAMA Network Open.*

eTable 6. Direct and Indirect Estimates From the Fixed-Effects Mantel-Haenszel NMA of Complications

eTable 7. Comparisons of the Direct and Indirect Estimates From the Fixed-Effects NMA of Reoperation

eTable 8. Direct and Indirect Estimates From the Random-Effects NMA of Recurrence

eTable 9. League Table of Pairwise Comparisons for Recurrence (Fixed-Effects Mantel-Haenszel)

eTable 10. Direct and Indirect Estimates From the Fixed-Effects Mantel-Haenszel NMA of Recurrence

eTable 11. CINEMA Assessments for the Primary Outcome

eAppendix. Search Results

This supplemental material has been provided by the authors to give readers additional information about their work.

eFigure 1. Study Selection Flowchart

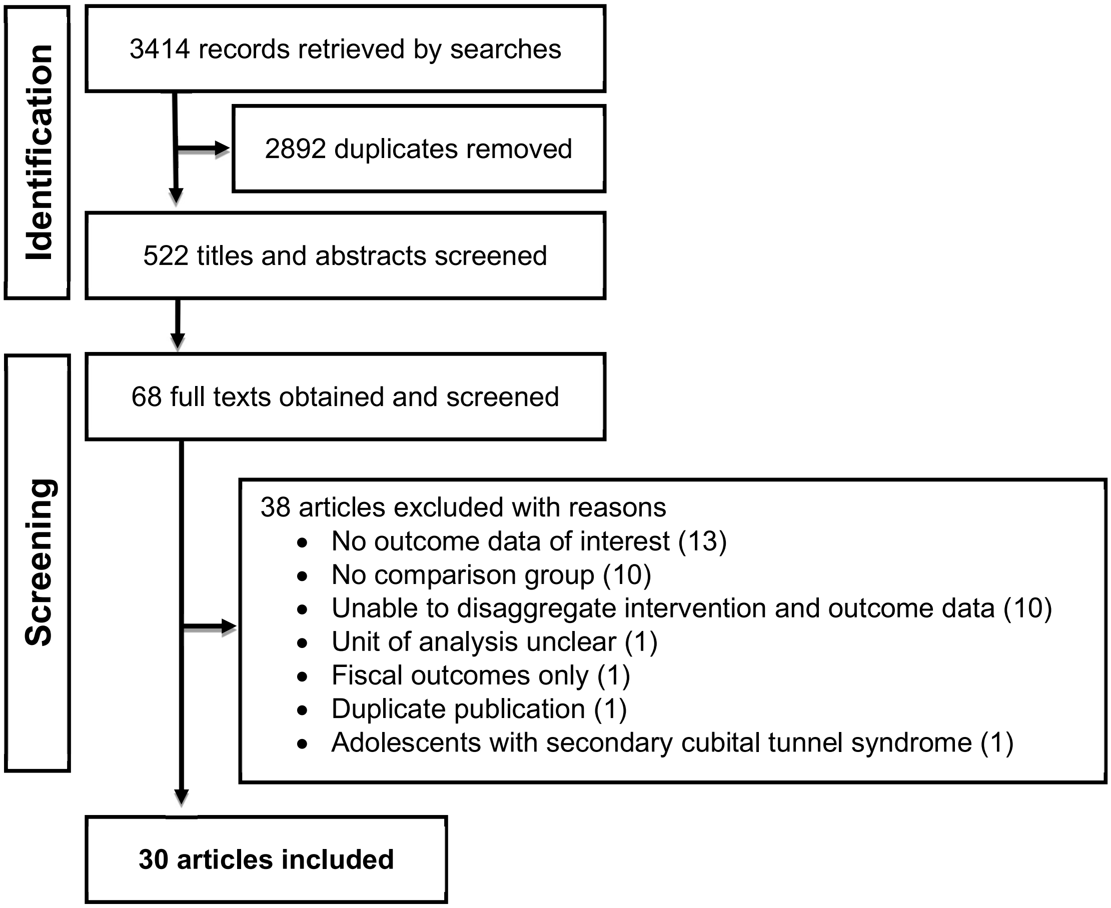

© 2020 Wade RG et al. *JAMA Network Open.*

eFigure 2. The Average Risk of Bias Contributions for Each Comparison

Treatment abbreviations as per Figure 1. Yellow is moderate risk whilst red is high risk.

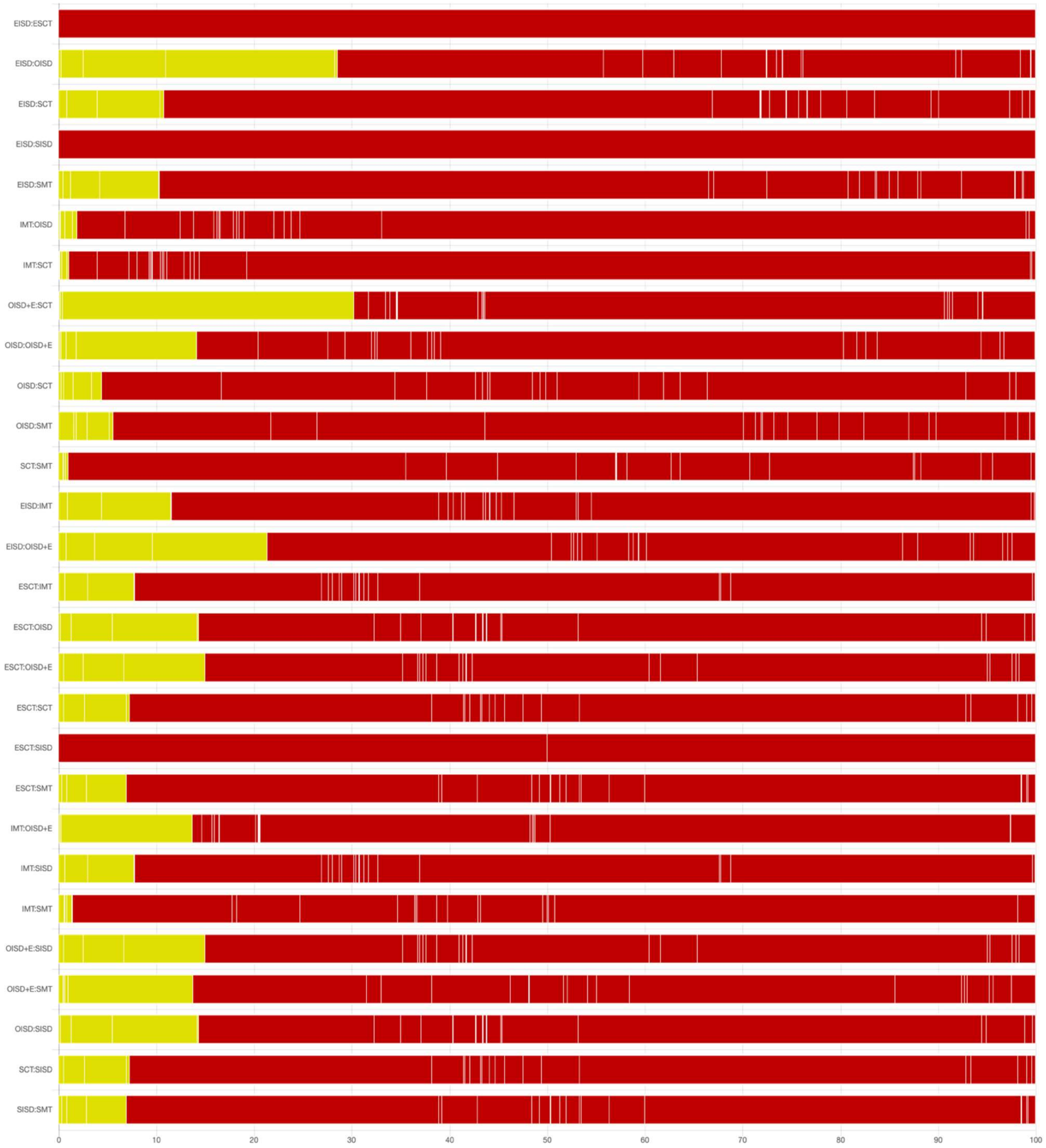

© 2020 Wade RG et al. *JAMA Network Open.*

eFigure 3. Risk of Bias Summary for Randomized Studies

	The randomisation process	Deviations from the assigned intervention	Failure of adherence to intervention	Missing outcome data	Measurement of the outcome	Selection of the reported results	Overall risk of bias
Bartels 2005	?	−	−	−	−	?	−
Biggs 2006	?	?	−	−	−	?	−
Gervasio 2005	?	+	+	−	+	?	?
Geutjens 1996	?	+	+	+	+	?	?
Heikenfeld 2013	?	?	−	+	−	?	?
Keiner 2009	?	?	?	−	−	?	?
Krejčí 2018	?	?	−	+	−	?	?
Schmidt 2015	?	+	?	+	+	?	?

© 2020 Wade RG et al. *JAMA Network Open.*

eFigure 4. Risk of Bias Summary for Nonrandomized Studies

	Confounding	Selection	Misclassification of the intervention	Deviations from the intended intervention	Missing data	Measurement of the outcomes	Selective reporting of the results	Overall risk of bias
Asamoto 2005	−	−	+	+	−	−	−	−
Bacle 2014	−	−	+	+	?	−	−	−
Baek 2006	−	−	+	+	?	−	−	−
Bimmler 1996	−	−	+	+	?	−	−	−
Bolster 2014	−	+	+	+	?	−	−	−
Capo 2011	−	−	+	+	?	−	−	−
Charles 2009	−	−	+	+	?	−	−	−
Dützmann 2013	−	−	+	+	?	−	−	−
Hahn 2010	−	−	+	+	+	−	−	−
Izadpanah 2015	−	−	+	+	?	?	−	−
Jaddue 2009	−	+	+	+	?	−	−	−
Kamat 2014	−	−	+	+	−	−	−	−
Kose 2007	−	−	+	+	?	−	−	−
Luo 2010	−	−	+	+	?	−	−	−
Martin 2014	−	−	+	+	?	−	−	−
Mitsionis 2010	−	−	+	+	?	−	−	−
Stuffer 1992	−	−	+	+	?	−	−	−
Teo 2010	−	?	+	+	+	−	−	−
Tong 2017	−	−	+	+	?	−	−	−
Watts 2009	−	−	+	+	?	−	−	−
Zhang 2017	−	−	+	+	?	−	−	−
Zhou 2012	−	?	+	+	?	?	−	−

© 2020 Wade RG et al. *JAMA Network Open.*

eFigure 5. Design-Adjusted Analyses for Response to Treatment

Results from the design-adjusted analysis, combining evidence from randomised (RCT) and non-randomised studies (NRS) for the primary outcome. The top estimate in each forest-plot is derived from RCT data only, each subsequent estimate is obtained from a NMA that down-weights non-randomised studies. The bottom estimate in each forest-plot corresponds to the naïve NMA, which includes all data from both randomised and non-randomised studies without any further adjustment. Abbreviations as per Figure 1.

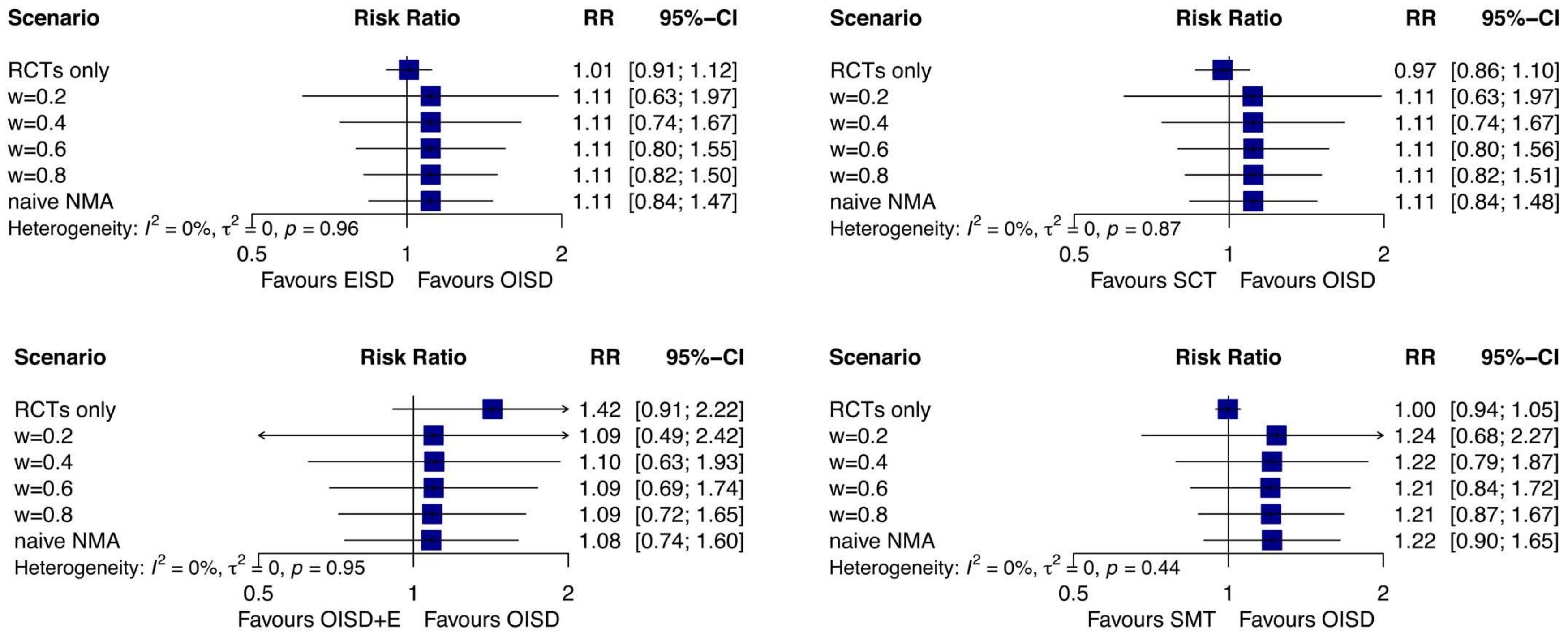

© 2020 Wade RG et al. *JAMA Network Open.*

eFigure 6. Forest Plots for Response to Treatment

Forest plots of the network estimates for the relative risk (RR) of symptomatic cure compared to open in-situ decompression. NMA estimates derived from randomised, non-randomised and all studies are shown. Abbreviations as per Figure 1.

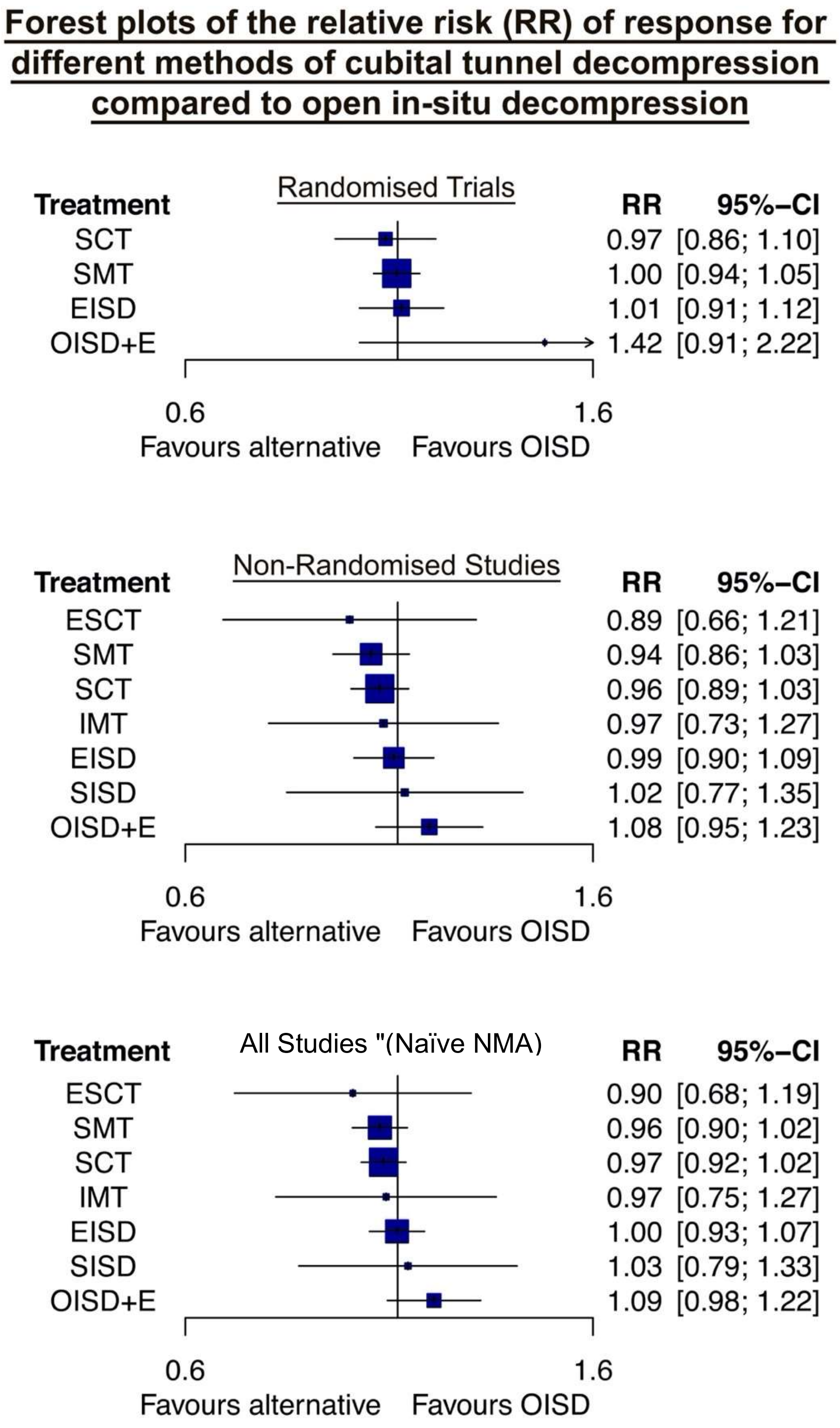

eFigure 7. Network Plot of Randomized Studies Included in Analysis for Response to Treatment

Network plot of interventions and direct comparisons from RCTs only, in the analysis of response to treatment. The size of the nodes correspond to the number of patients, the thickness of the connecting lines corresponds to the number of studies and the colour of the lines corresponds to the average risk of bias assessment (yellow = unclear or moderate risk, red = high risk). NRS = non-ransomised studies, RCT = randomised controlled trias. Abbreviations as per Figure 1.

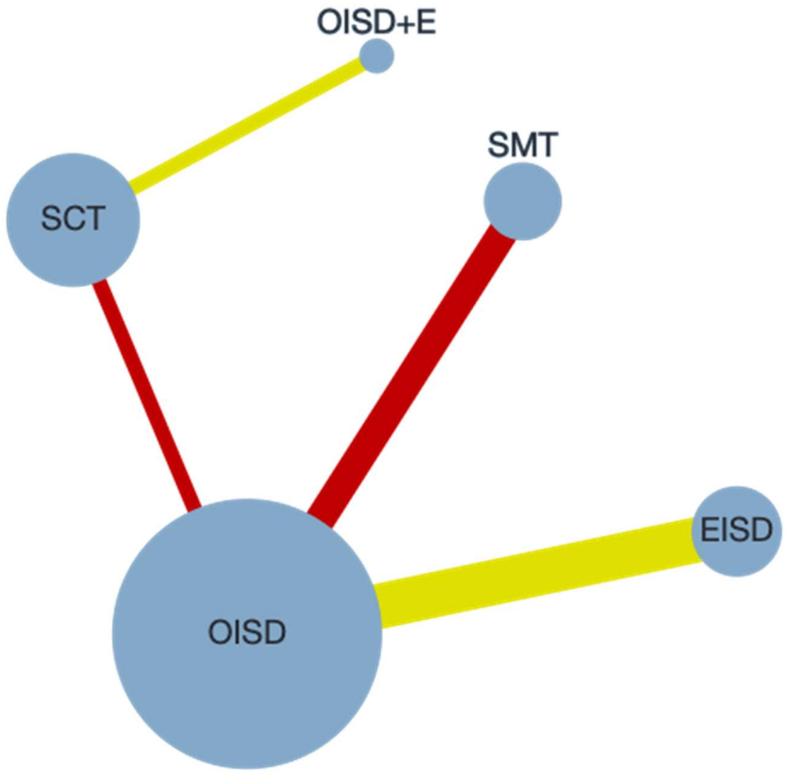

© 2020 Wade RG et al. *JAMA Network Open.*

eFigure 8. Network Plot of Nonrandomized Studies Included in Analysis for Response to Treatment

Network plot of interventions and direct comparisons from NRSs only, in the analysis of response to treatment. The size of the nodes correspond to the number of patients, the thickness of the connecting lines corresponds to the number of studies and the colour of the lines corresponds to the average risk of bias assessment (yellow = unclear or moderate risk, red = high risk). NRS = non-ransomised studies, RCT = randomised controlled trias. Abbreviations as per Figure 1.

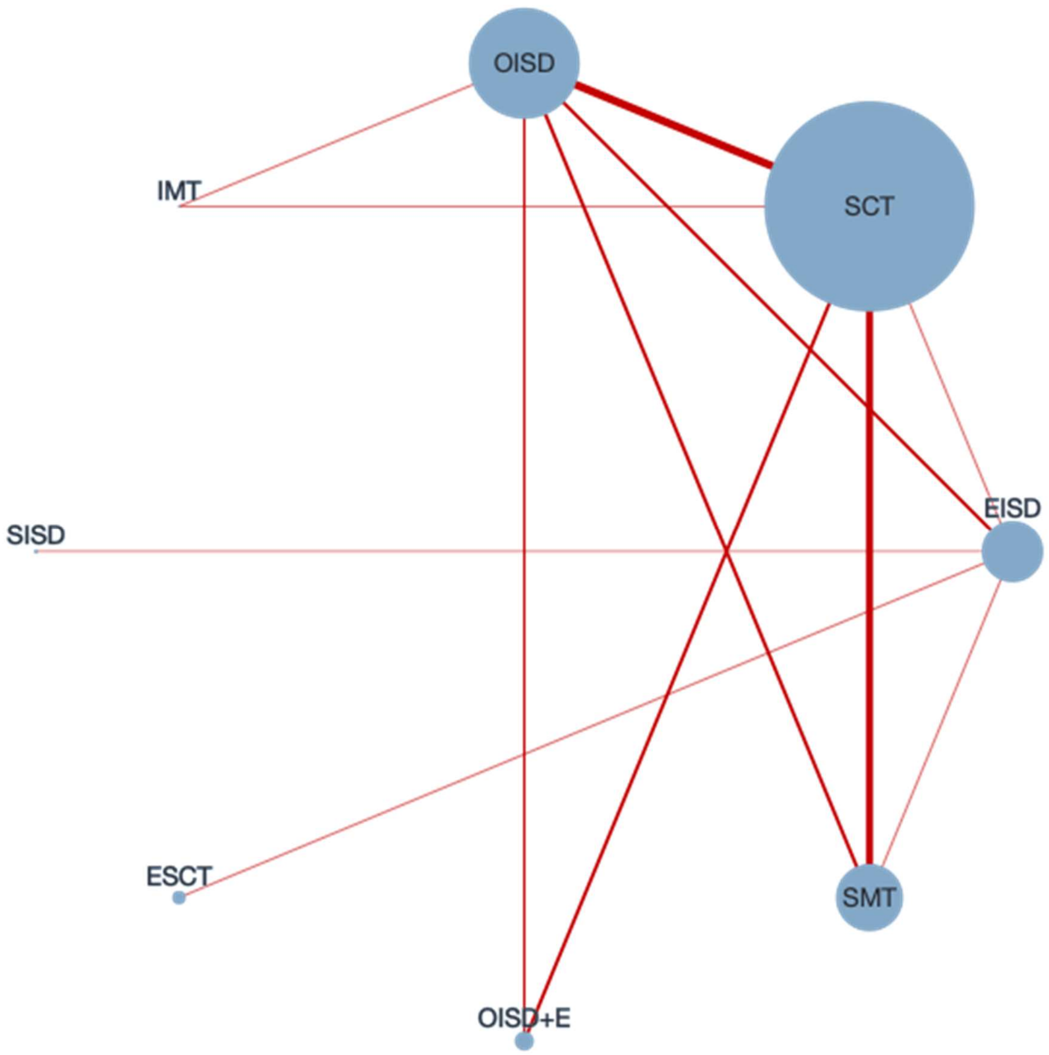

© 2020 Wade RG et al. *JAMA Network Open.*

eFigure 9. Network Heat Plot for Response to Treatment

Network heat plot for response to treatment showing a red hotspot of inconsistency between direct and indirect evidence for the naïve network of the primary outcome response to treatment. Abbreviations as per Figure 1.

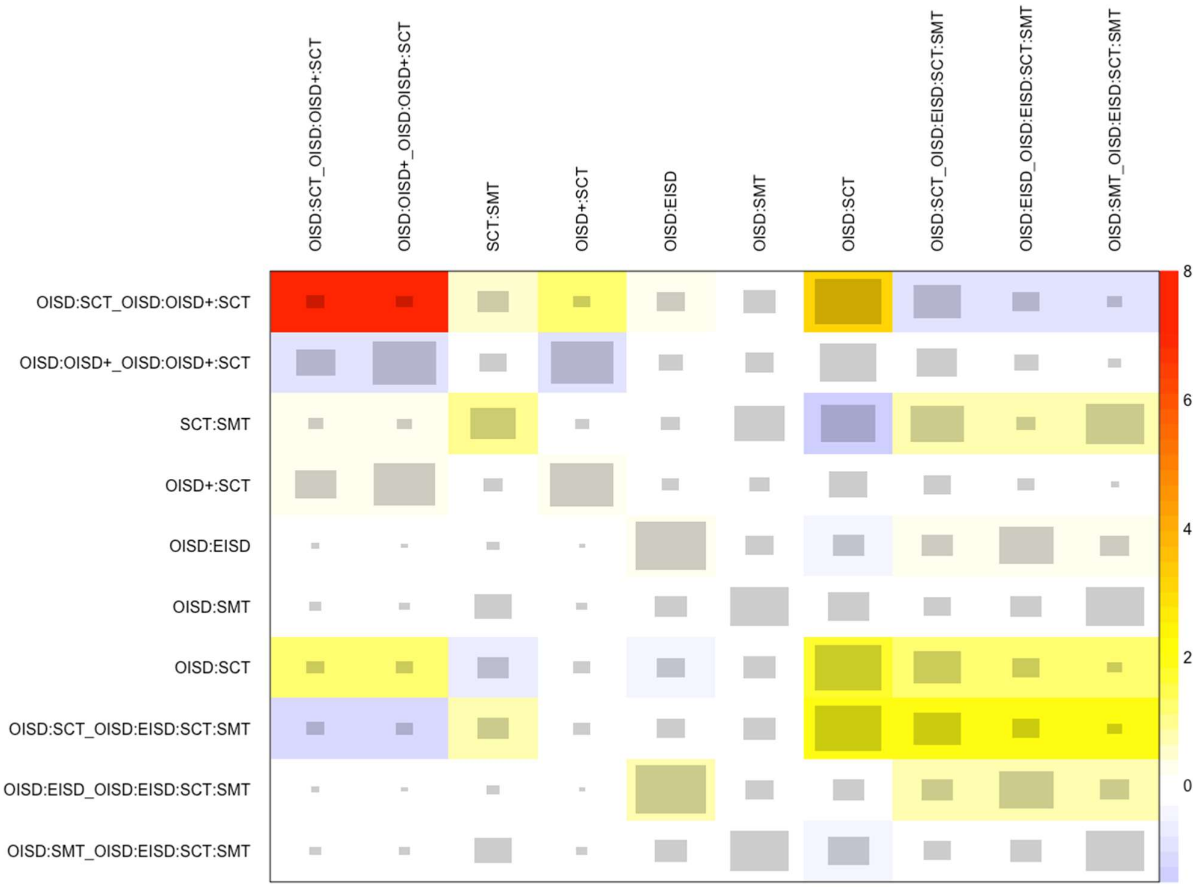

© 2020 Wade RG et al. *JAMA Network Open.*

eFigure 10. Network Plot of Studies Included in the Analysis of Complications

Network plot of interventions and direct comparisons in the analysis of complications. The size of the nodes correspond to the number of patients, the thickness of the connecting lines corresponds to the number of studies and the colour of the lines corresponds to the average risk of bias assessment (yellow = unclear or moderate risk, red = high risk). NRS = non-ransomised studies, RCT = randomised controlled trias. Abbreviations as per Figure 1.

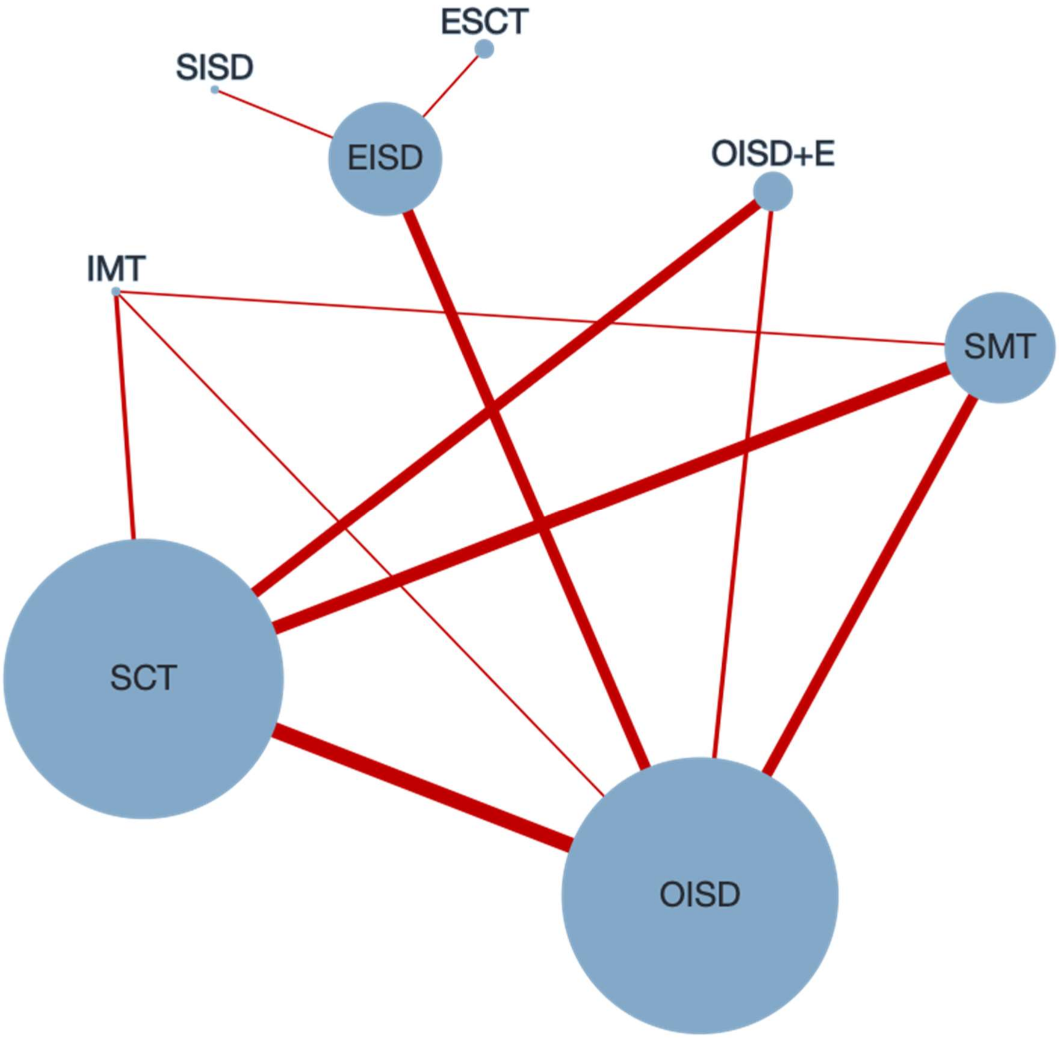

eFigure 11. Network Heat Plot for Complications (Naive Random-Effects NMA)

Network heat plot for complications showing no inconsistency between direct and indirect evidence for the naïve random-effects NMA of complications. Abbreviations as per Figure 1.

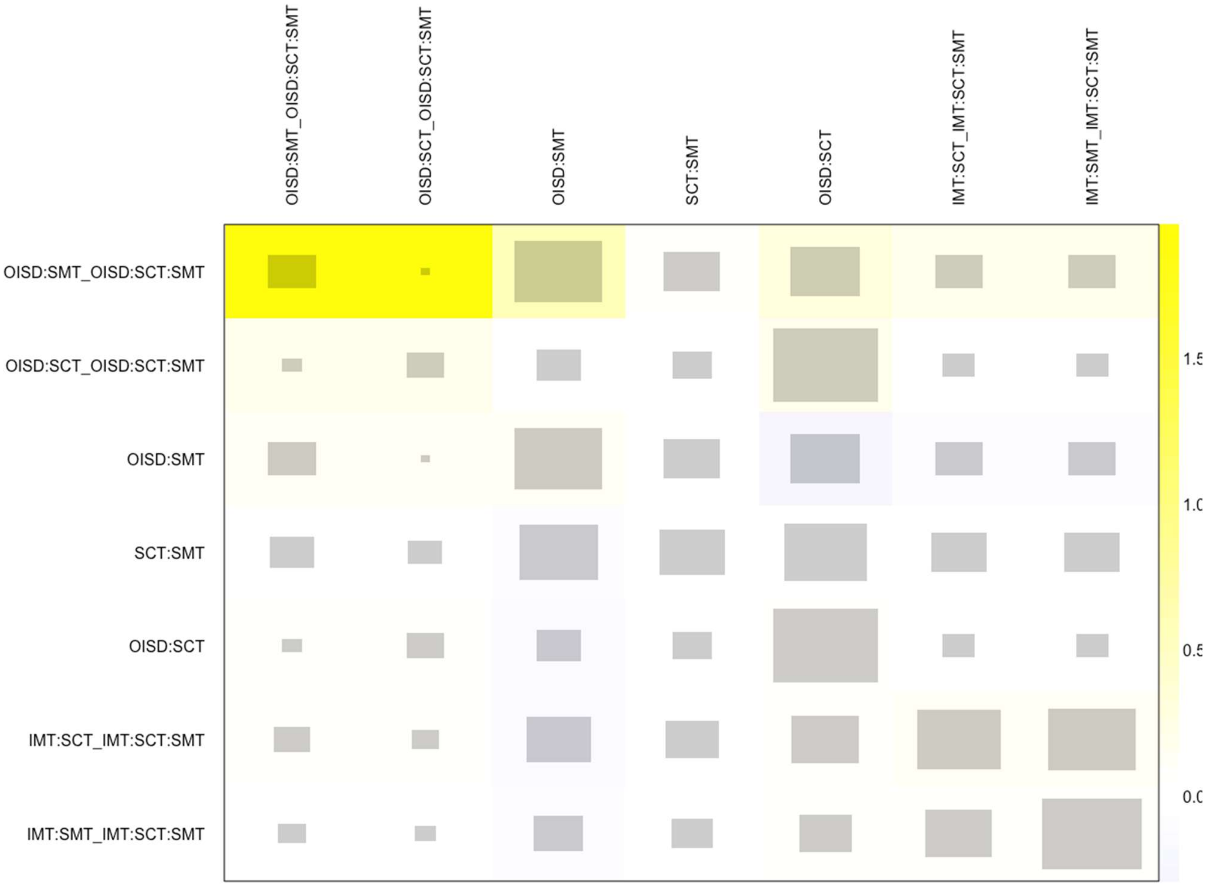

© 2020 Wade RG et al. *JAMA Network Open.*

eFigure 12. Network Heat Plot for Complications (Naive Fixed-Effects NMA)

Network heat plot for complications showing no inconsistency between direct and indirect evidence for the naïve fixed-effects NMA of complications. Abbreviations as per Figure 1.

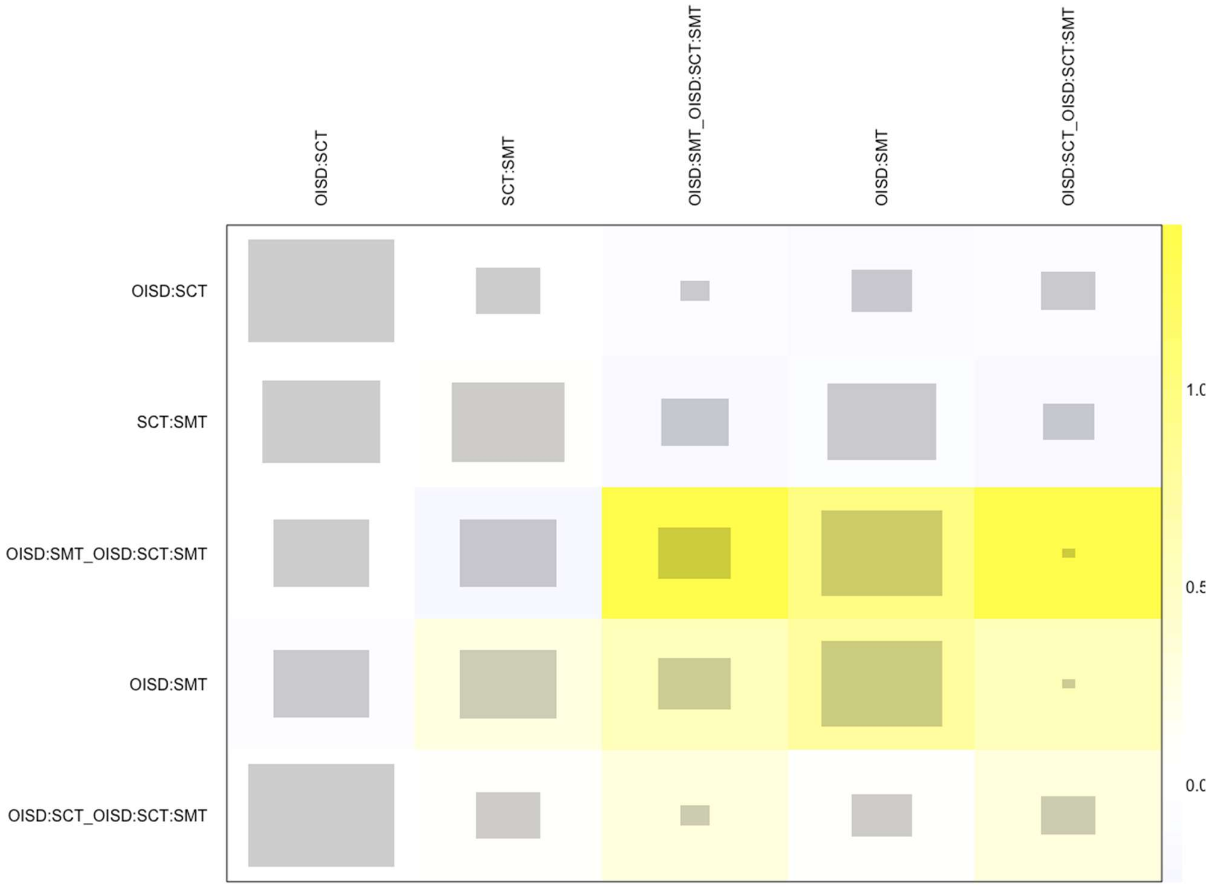

© 2020 Wade RG et al. *JAMA Network Open.*

eFigure 13. Network Plot of Studies Included in the Analysis of Reoperation

Network plot of interventions and direct comparisons in the analysis of reoperation. The size of the nodes correspond to the number of patients, the thickness of the connecting lines corresponds to the number of studies and the colour of the lines corresponds to the average risk of bias assessment (yellow = unclear or moderate risk, red = high risk). NRS = non-ransomised studies, RCT = randomised controlled trias. Abbreviations as per Figure 1.

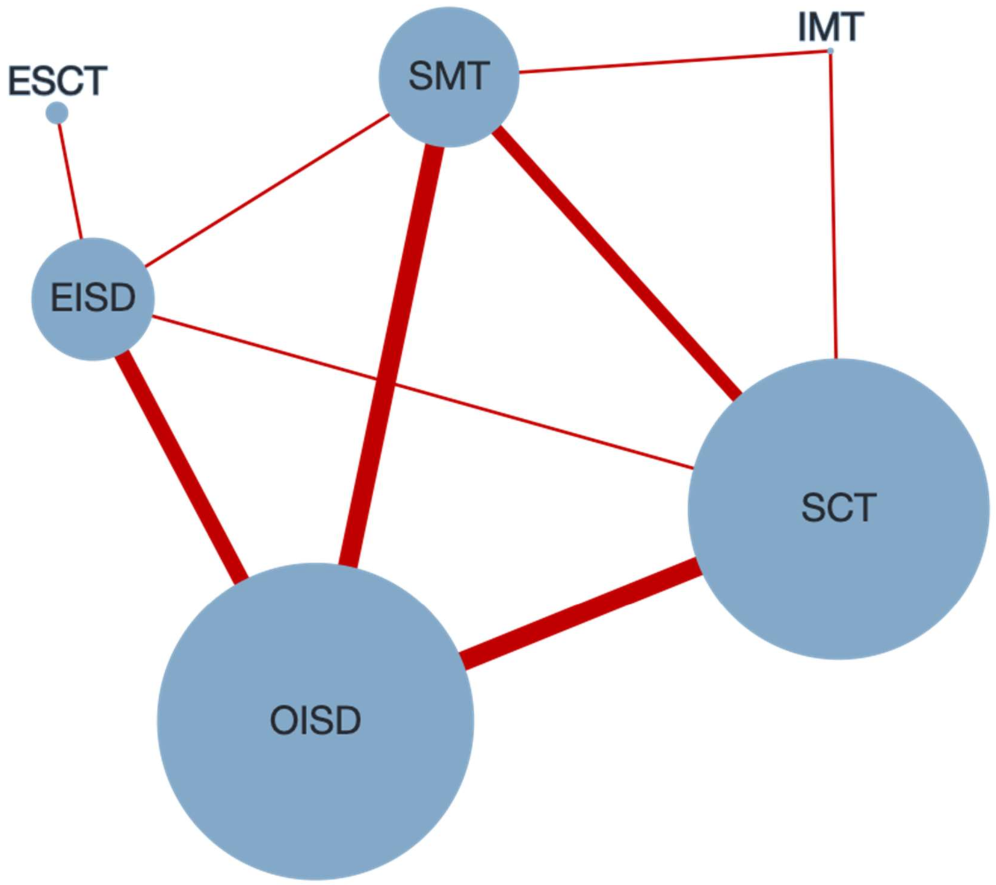

© 2020 Wade RG et al. *JAMA Network Open.*

eFigure 14. Network Heat Plot for Reoperation (Naive Fixed-Effects Mantel-Haenszel NMA)

Network heat plot showing no inconsistency between direct and indirect evidence for the naïve fixed-effects Mantel-Haenszel NMA of reoperation. Abbreviations as per Figure 1.

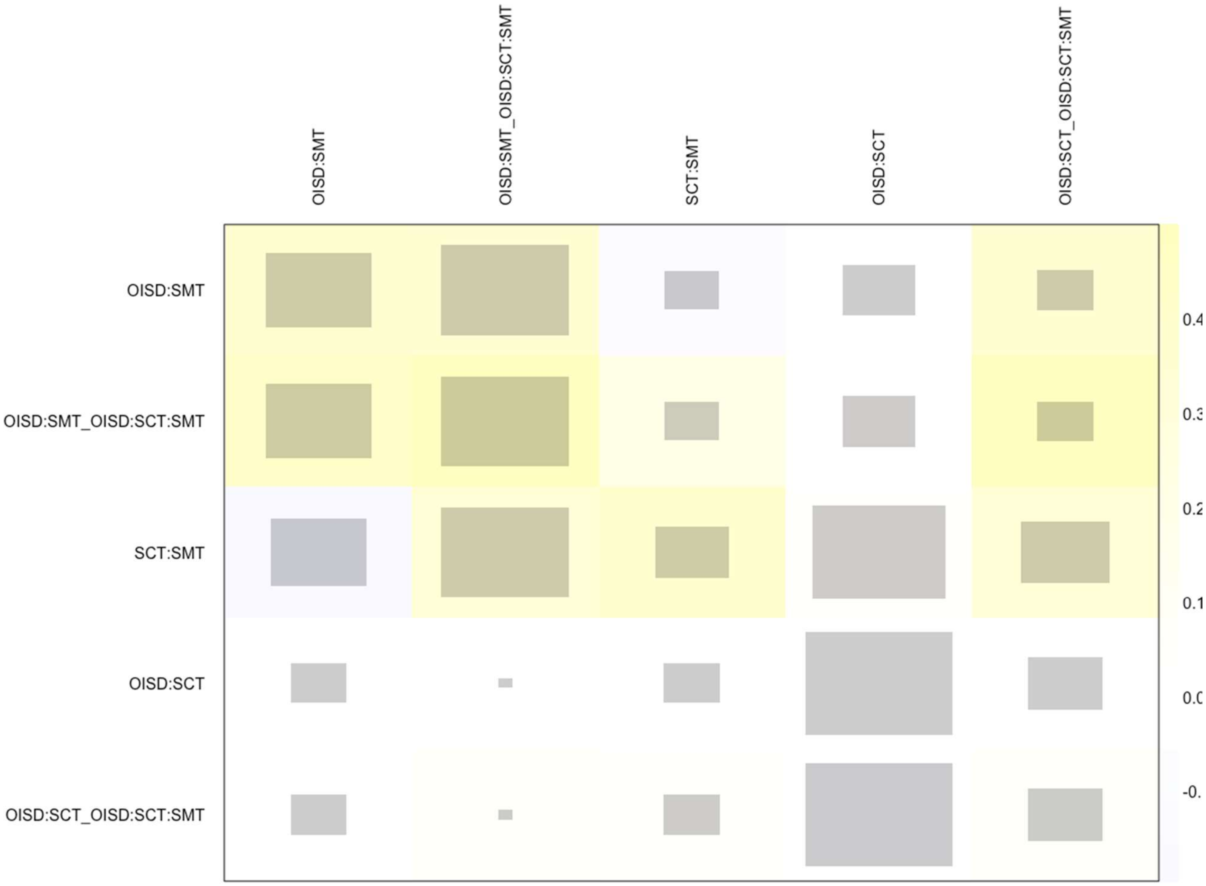

© 2020 Wade RG et al. *JAMA Network Open.*

eFigure 15. Network Plot of Studies Included in the Analysis of Recurrence

Network plot of interventions and direct comparisons in the analysis of recurrence. The size of the nodes correspond to the number of patients, the thickness of the connecting lines corresponds to the number of studies and the colour of the lines corresponds to the average risk of bias assessment (yellow = unclear or moderate risk, red = high risk). NRS = non-randomised studies, RCT = randomised controlled trias. Abbreviations as per Figure 1.

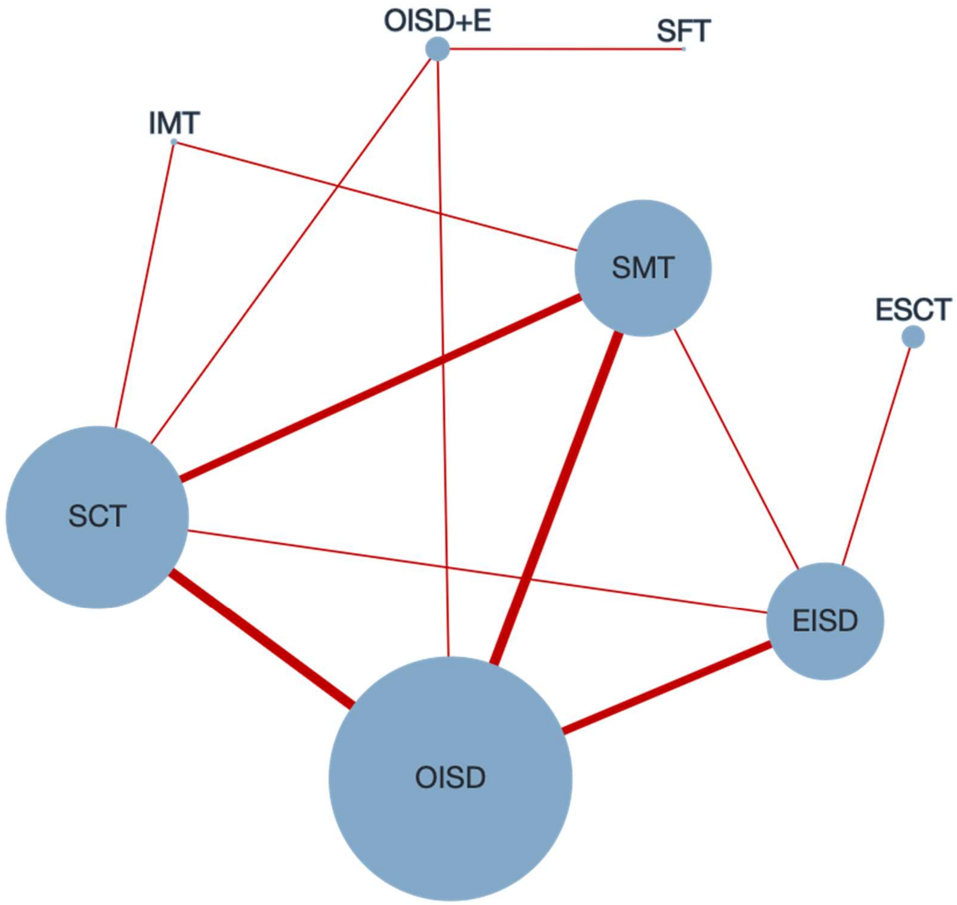

© 2020 Wade RG et al. *JAMA Network Open.*

eFigure 16. Network Heat Plot for Recurrence (Naive Random-Effects NMA)

Network heat plot showing no inconsistency between direct and indirect evidence for the naïve random-effects NMA of recurrence. Abbreviations as per Figure 1.

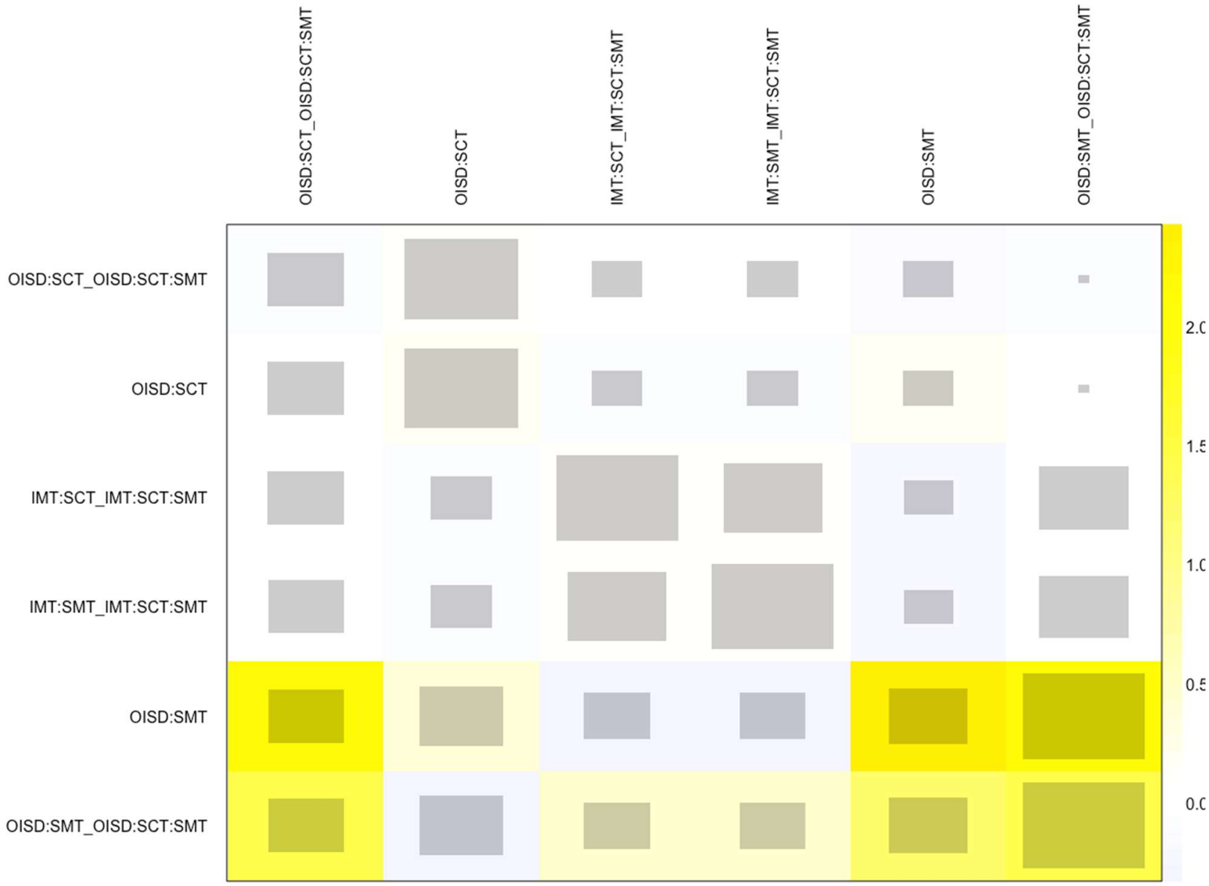

© 2020 Wade RG et al. *JAMA Network Open.*

eFigure 17. Network Heat Plot for Recurrence (Naive Fixed-Effects Mantel-Haenzel NMA)

Network heat plot showing no inconsistency between direct and indirect evidence for the naïve fixed-effects Mantel-Haenzel NMA of recurrence. Abbreviations as per Figure 1.

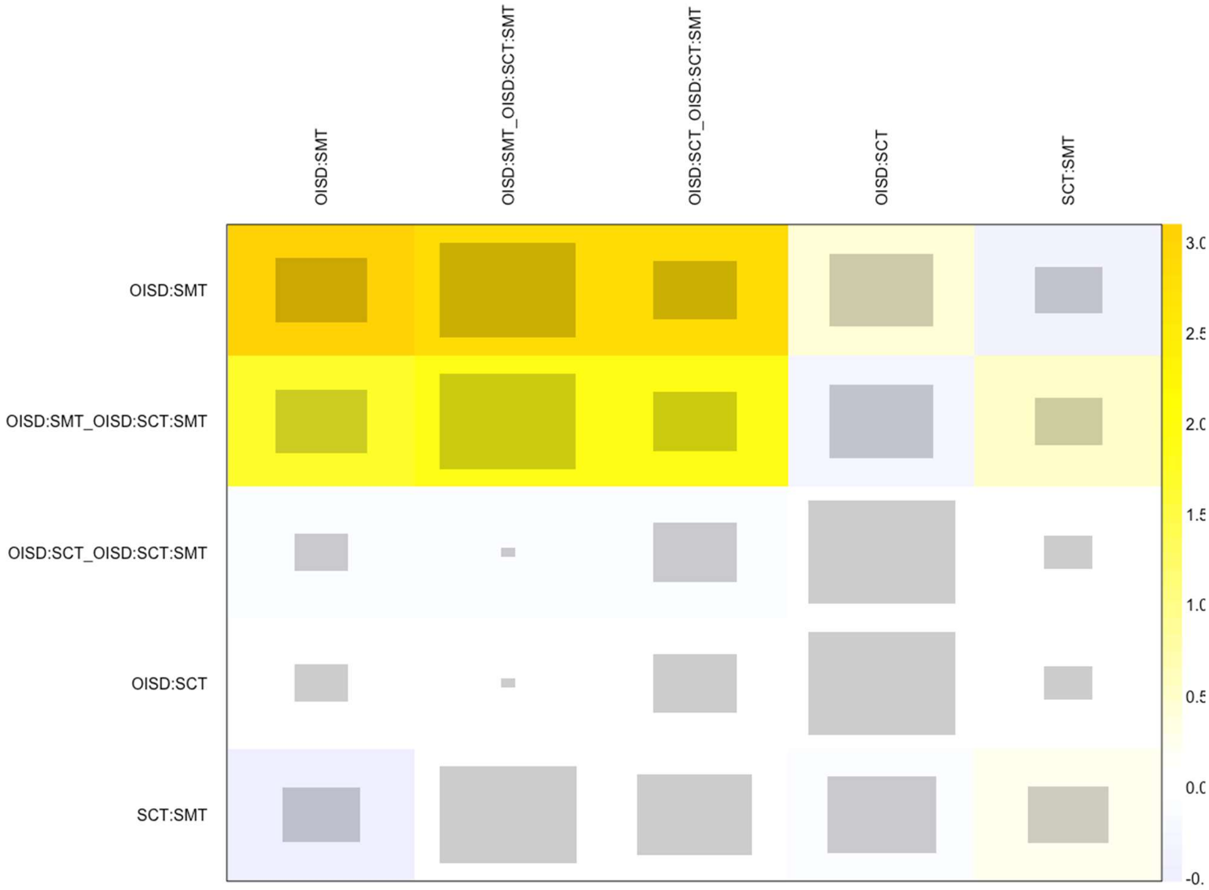

© 2020 Wade RG et al. *JAMA Network Open.*

eFigure 18. Comparison-Adjusted Funnel Plot

A comparison-adjusted funnel plot which is symmetrical and thus, suggests the absence of small study effects (SSE) for the primary outcome of response to treatment. To construct the funnel plot in a meaningful way, it is required to determine the expected direction of SSE in each pairwise comparison in the network; we hypothesised the SSE would be largest in descending order as follows: minimally invasive surgery > endoscopic procedures > transpositions > in-situ decompressions. Abbreviations are as per Figure 1.

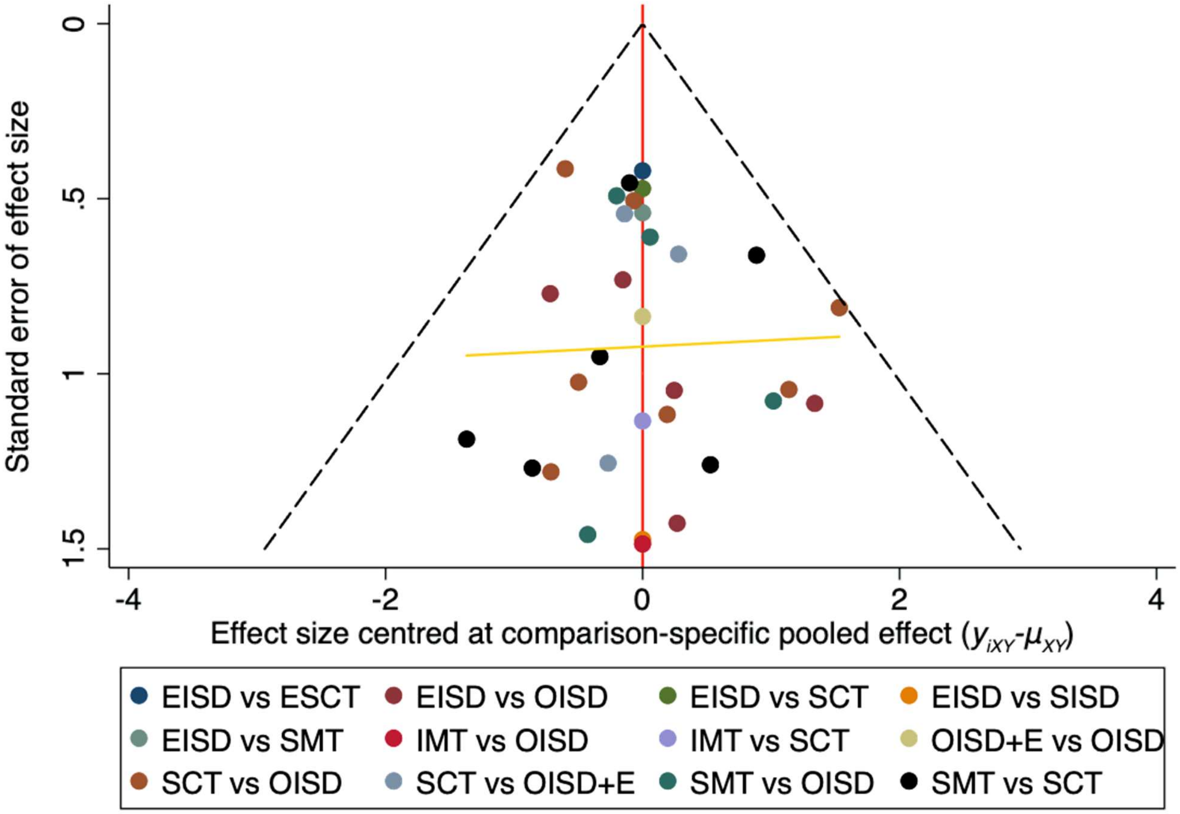

© 2020 Wade RG et al. *JAMA Network Open.*

eTable 1. Summary of Study Characteristics

Study ID	Study Design	Location	Interventions (Recruited $N_{patients}$:N_{limbs} | M:F)	Months with symptoms	Preoperative Assessment scale	Preoperative symptom severity	Definition of response to treatment and scale used	Follow up (FU); attrition
Asamoto 2005	Retrospective cohort study	Japan	Open in-situ decompression (13:unclear | unclear)	Not described	Not described	Not described	Study specific outcome measure: Responder = “excellent” or “good”; Non-responder = “no change” or “poor”	Mean 2.2 months (range 1 to 24); 9 patients lost to follow-up
			Subcutaneous transposition (58:unclear | unclear)					
			Intramuscular transposition (10:unclear | unclear)					
Bacle 2014	Retrospective cohort study	France	Open in-situ decompression (48:48 | unclear)	Mean 8	McGowan-Goldberg	I=20, II=53, III=27	Study specific outcome measure: Responder: “improved”; Non-responders: “worse or unchanged”	Mean 7.6 years (Range 9-144); 103 lost to FU (4 OISD, 51 SCT, 48 EISD)
			Endoscopic in-situ decompression (143:151 | unclear)	Mean 28		I=48, II=44, III=15		
			Subcutaneous transposition (229:253 | unclear)	Mean 25		I=45, II=34, III=21		
			Submuscular transposition (82:84 | unclear)	Mean 13		I=52, II=44, III=4		
Baek 2006	Not described	Australia	Open in-situ decompression and medial epicondylectomy (22:22:15:7)	Mean 20	McGowan-Goldberg	I=4, II=12, III=6	Study specific outcome measure: Responder: “fair, good or excellent” Non-responders: “poor”	1 year; none lost to FU
			Subcutaneous transposition (34:34: 24:10)	Mean 28		I=3, II=21, III=10		
Bartels 2005	RCT	Netherlands	Open in-situ decompression (75:75 | 46:29)	Mean 8.6 (SD 10.1)	Bespoke scale	II=21, III=54	Any improvement on a study-specific scale 7-point Likert scale	12 months;<br>5 lost to FU (2 OISD, 3 SCT)
			Subcutaneous transposition (77:77 | 48:29)	Mean 8.9 (SD 10.4)		I=1, II=19, III=57		
Biggs 2006	Quasi-RCT	Australia	Open in-situ decompression (23:unclear | 16:7)	Not described	McGowan (and LSUMC)	I=6, II=13, III=4	Any improvement in the McGowan score	OISD Mean 3.23 years FU, SCT mean 3.5 years FU; 3 lost to FU but unclear which group(s)
			Submuscular transposition (21:unclear | 17:4)			I=2, II=14, III=5		
Bimmler 1996	Not described	France	Open in-situ decompression (31:unclear | unclear)	Not described	McGowan	I=9, II=10, III=12	Bishop scale; responder = “excellent” or “good”; Non-responder = “fair”	6.3 years; attrition not described
			Submuscular transposition (48:unclear | unclear)			I=18, II=20, III=10		
Bolster 2014	Not described	Netherlands	Endoscopic in-situ decompression (20:20 | 9:11)	Mean 15.7 (SD 16.8)	Dellon	I=3, II=5, III=3, 9=unknown	Bishop scale Responder = “excellent” or “good”; Non-responder = “fair”	EISD mean 7.1 months FU, SISD mean 6.7 months; 18 lost to FU (12 EISD, 6 SISD)
			Speculum in-situ decompression (22:22 | 11:11)	Mean 20.8 (SD 26.8)		I=4, II=11, III=1, 6=unknown		
Capo 2011	Retrospective cohort study	USA	Subcutaneous transposition (9:9 | unclear)	Not described	A “standardized questionnaire”	Not described	Any improvement on their “standardized questionnaire”	Mean 14 months (+/- 5); attrition not described

© 2020 Wade RG et al. *JAMA Network Open.*

			Open in-situ decompression and epicondylectomy (9:9 | unclear)					Mean 21 months (+/- 7); attrition not described
Charles 2009	Retrospective cohort study	France	Subcutaneous transposition (24:24 | 19:5)	Mean 12 (range 2, 48)	McGowan	II=14, III=10	Responder = any improvement in the McGowan score as according to two independent assessors	Mean 3 years (range 2-5 years); attrition not described
			Submuscular transposition (25:25 | 18:7)	Mean 12 (range 2, 48)		II=11, III=14		Mean 7 years (range 2-11); attrition not described
Dützmann 2013	Retrospective cohort study	Germany	Open in-situ decompression (59:unclear | 34:25)	Not described	McGowan	I=8, II=16, III=35	Bishop scale; responder = "excellent" or "good"; Non-responder = "fair"	2 years; attrition not described
			Endoscopic in-situ decompression (55:unclear | 29:26)			I=7, II=15, III=33		
Gervasio 2005	RCT	Italy	Open in-situ decompression (35:unclear | 25:10)	Not described	Dellon	Not described	Bishop scale; responder = "excellent" or "good"; Non-responder = "fair"	Mean 3.9 years; attrition not described
			Submuscular transposition (35:unclear | 23:12)					Mean 3.9 years; attrition not described
Geutjens 1996	RCT	UK	Open in-situ decompression and epicondylectomy (25: unclear | unclear)	Not described	No scale used; clinical parameters reported	Not described	Patient opinion; responder = "cured" or "better", non-responder = "same" or "worse"	Mean 4.5 years; 9 lost to FU (4 untraceable records, 2 deaths, 3 moved away)
			Subcutaneous transposition (22: unclear | unclear)					
Hahn 2010	Retrospective cohort study	Korea	Open in-situ decompression and epicondylectomy (29:unclear | 17:12)	Mean 17.3 (range 3-96)	Dellon	I=2, II=15, III=12	Modified Bishop score; responder = "excellent" or "good"; Non-responder = "fair" or "poor"	Mean 2.6 years (range 24-39); attrition not described
			Subcutaneous transposition (27:unclear | 17:10)	Mean 19.1 (range 3-60)		I=1, II=15, III=11		Mean 3.4 years (range 24-52 months); attrition not described
Heikenfeld 2013	RCT (abstract)	Germany	Open in-situ decompression (15:unclear | unclear)	Not described	Dellon	Not described	Modified Bishop score; responder = "very good" "good" or "satisfactory"	12 moths; 2 lost to FU
			Endoscopic in-situ decompression (15:unclear | unclear)					12 months; 1 lost to FU
Izadpanah 2015	Retrospective cohort study (abstract)	USA	Open in-situ decompression (40:unclear | unclear)	Mean 12.1 (SD 5.8; range 3-60)	McGowan	Not described	Not described	Minimum 12 months; attrition not described
			Subcutaneous transposition (48:unclear | unclear)					
Jaddue 2009	Prospective cohort study	Iraq	Subcutaneous transposition (13:unclear | 10:3)	Not described	Dellon	II=13	Bishop score; responder = "excellent" or "good"; Non-responder = "poor"	12 months; attrition not described
			Submuscular transposition (13:unclear | 10:3)			II=13		
Kamat 2014	Retrospective cohort study	New Zealand	Open in-situ decompression (179:unlear | 84:95)	Mean 15 (range 6-23)	McGowan	I=92, II=56, III=31	McGowan; responder = any improvement	3 months; attrition not described
			Subcutaneous transposition (301:unclear | 101:200)			I=191, II=80, III=30		
Keiner 2009	Prospective cohort study	Germany	Open in-situ decompression (16:16 | 12:5)	Mean 8.4 (range 1.5-36)	McGowan	I=4, II=6, III=6	McGowan; telephone interview by an independent healthcare	4.3 years (range 38-66 months); 7 lost to follow-up but group unclear

			Submuscular transposition (17:17 | 1:1)	Mean 13 (range 2-84)		I=11, II=2, III=4	professional. Responder = any improvement	5.3 years (range 41-73 months); 7 lost to follow-up but group unclear
Köse 2007	Retrospective cohort study	Turkey	Submuscular transposition (16:16 | unclear)	Median 20 (range 3-36)	McGowan	I=8, II=4, III=4	Wilson-Kraut; responder = “excellent”, “good” or “fair”. Non-responder = “poor”	Mean 2.3 years (SD 21 months); 1 lost to FU but unclear from which group
			Subcutaneous transposition (18:18 | unclear)	Median 15 (range 3-24)		I=10, II=5, III=3		
			Intramuscular transposition (15:15 | unclear)	Median 18 (range 1-24)		I=8, II=5, III=2		
Krejčí 2018	RCT	Czech Republic	Open in-situ decompression (23:23 | 12:11)	>6 weeks	McGowan	Mean 2.74	Modified Bishop score; responder = “excellent”, “good” or “fair”; non-responder = “poor”	12 months; no attrition
			Endoscopic in-situ decompression (20:20 | 11:11)			Mean 2.5		12 months; 2 lost to FU
Luo 2010	Not described	China	Subcutaneous transposition (24:unclear | 8:8)	Mean 18 months (4 months – 6 years)	McGowan	“All patients were Stage II or III”	Bishop score; responder = “excellent” or “good”; Non-responder = “poor”	1-3 years; attrition not described
			Submuscular transposition (42:unclear | 30:12)	Mean 15 months (range 3 months – 5.5 years)				
Martin 2014	Retrospective cohort study	Germany	Endoscopic in-situ decompression (55:58 | unclear)	“At least 2-3 months”	Not described	Not described	Modified Bishop score; responder = “excellent”, “good” or “fair”; non-responder = “poor”	Up to 2 years; attrition not described
			Endoscopic subcutaneous transposition (52:52 | unclear)					
Mitsionis 2010	Retrospective cohort study	Greece	Open in-situ decompression (34:34 | unclear)	Mean 15 months (range 2-48)	McGowan	I=2, II=25, III=4, 3=unknown	Wilson-Kraut; responder = “excellent”, “good” or “fair”. Non-responder = “poor”	24 months; 6 lost to FU
			Open in-situ decompression and epicondylectomy (46:46 | unclear)			II=33, III=12, 1=unknown		
			Subcutaneous transposition (39:39 | unclear)			II=22, III=15, 2=unknown		
Schmidt 2015	RCT	Germany	Endoscopic in-situ decompression (unclear:29 | 17:12)	Mean 14 months (±20)	McGowan	II=21, III=8	Modified Bishop score; responder = “excellent”, “good” or “fair”; non-responder = “poor”	16.8 months; 2 lost to FU
			Open in-situ decompression (unclear:27 | 16:11)	Mean 20 months (±24)		I=1, II=15, III=11		16.8 months; 1 lost to FU)
Stuffer 1992	Not described	Australia	Subcutaneous transposition (33:unclear | unclear)	Not described	Bespoke scale (good, satisfactory, moderate, poor)	Not described	Bespoke scale (responder = “good”, “satisfactory” or “moderate”; non-responder = poor)	Mean 9.6 years (±3.6); minimum 2 years, maximum 17 years
			Submuscular transposition (18:unclear | unclear)					
Teo 2010	Retrospective cohort study	UK	Open in-situ decompression (2:2 | 1:1)	Mean 5 months	McGowan	I=2	McGowan; responder = any improvement	Median 3 months (IQR 1-3); none lost to FU
			Subcutaneous transposition (49:49 | 37:12)	Mean 15 months		I=8, II=36, III=5		Median 3 months (IQR 2-6); 1 lost to FU (died)
			Open in-situ decompression and epicondylectomy (2:2 | 2:0)	Mean 6 months		I=1, II=1		Median 5.5 months (IQR 4-7); none lost to FU
Tong 2017	Retrospective	China	Open in-situ decompression (unclear:5 | unclear)		McGowan	III= 5		

© 2020 Wade RG et al. *JAMA Network Open.*

			Subcutaneous transposition (unclear:21| unclear)	Mean 10.3 months (range 1-48)		III = 21	Modified Bishop score; responder = “excellent”, “good” or “fair”; non-responder = “poor”	Mean 56.2 months (range 24-121)
Watts 2009	Prospective cohort study	Australia	Endoscopic in-situ decompression (19:19 | 13:6)	Not described	McGowan	I=10, II=5, III=4	Patient satisfaction; responder = “satisfied”; non-responder = “dissatisfied”	12 months; 2 lost to FU
			Open in-situ decompression (15:15 | 6:10)			I=4, II=6, III=5		12 months; 1 lost to FU
Zhang 2017	Retrospective cohort study	USA	Open in-situ decompression (147:157 | unclear)	Not described	McGowan	I=80, II=54, III=13	Not described	Median 10 months (range 3-72); losses to FU not described
			Subcutaneous transposition (unclear:29 | unclear)			Not described		Median 13 months (range 6-66); losses to FU not described
			Submuscular transposition (unclear:61 | unclear)			Not described		Median 13 months (range 6-66); losses to FU not described
Zhou 2012	Not described	China	Subcutaneous transposition (20:20 | 12:8)	Mean 4.6 months (range 1-8)	Dellon	I=2, II=18	The Disability of the Arm, Shoulder and Hand Questionnaire; responder = any improvement	Mean 28 months (range 24-36); losses to FU not described
			Submuscular transposition (19:19 | 14:5)	Mean 4.8 months (range 1-7)		I=2, II=17		

*Grading scale for symptoms: I=mild, II=moderate (and includes McGowan IIA/IIB), III=Severe

LSUMC Louisiana State University Medical Centre classification system

© 2020 Wade RG et al. *JAMA Network Open.*

eTable 2. Summary of Variables That Might Moderate the Relative Effects of Treatments

Summary of clinical and methodological variables that might moderate the relative effects of the treatments**.** Abbreviations as per Figure 1.

**Variables**		**OISD**	**OISD+E**	**SCT**	**IMT**	**SMT**	**EISD**	**ESCT**	**SISD**	**p-value**¥
Median age in years (IQR)		48 (41, 53)	42 (28, 51)	47 (43, 49)	48 (48, 48)	50 (40, 56)	50 (29, 52)	*	50 (50, 50)	0.779
Percentage males; median (IQR)		58 (50, 63)	40 (28, 51)	62 (51, 71)	*	72 (66, 77)	68 (58, 71)	*	73 (73, 73)	0.664
Duration of symptoms in months; median (IQR)		9 (8, 20)	17 (6, 20)	17 (12, 22)	*	13 (13, 15)	16 (14, 28)	*	21 (21, 21)	0.664
Preoperative McGowan Grade; median (IQR)	I	26 (5, 48)	11 (4, 57)	11 (1, 25)	53 (53, 53)	17 (5, 43)	27 (12, 46)	*	27 (27, 27)	0.844
	II	32 (19, 56)	64 (53, 84)	44 (27, 58)	33 (33, 33)	40 (18, 43)	43 (27, 45)	*	73 (73, 73)	0.464
	III	36 (17, 60)	35 (13, 45)	17 (10, 38)	13 (13, 13)	27 (16, 44)	27 (21, 28)	*	7 (7, 7)	0.676

**No arm-specific data in the original studies*

¥*Unadjusted p-values from Dunn's test (Stata package dunntest). After adjustment for multiplicity using the Holm method, most pair-wise comparisons yielded p-values equal to 1 with a minority taking values >0.5; the presentation of p-values from the entire matrix of possible pairwise comparisons is impractical so please refer to the raw data if detailed information is desired.*

© 2020 Wade RG et al. *JAMA Network Open.*

eTable 3. Direct and Indirect Estimates From the Random-Effects NMA of Response to Treatment

Comparisons of the direct and indirect estimates from the random-effects NMA of response to treatment using the back-calculation method. Abbreviations as per Figure 1.

comparison	k	prop	direct	95%-CI	indir.	95%-CI	RoR	95%-CI	z	p-value
EISD:ESCT	1	1.00	1.1123	[0.8440; 1.4659]	.	.	.	.	.	.
EISD:IMT	0	0	.	.	1.0055	[0.7643; 1.3227]	.	.	.	.
EISD:OISD	6	0.91	0.9945	[0.9287; 1.0649]	0.9783	[0.7868; 1.2165]	1.0165	[0.8089; 1.2774]	0.14	0.8883
EISD:OISD+E	0	0	.	.	0.9101	[0.8023; 1.0325]	.	.	.	.
EISD:SCT	1	0.38	1.0241	[0.9033; 1.1610]	1.0278	[0.9309; 1.1349]	0.9964	[0.8492; 1.1691]	-0.04	0.9644
EISD:SISD	1	1.00	0.9740	[0.7562; 1.2547]	.	.	.	.	.	.
EISD:SMT	1	0.45	0.9666	[0.8541; 1.0938]	1.0553	[0.9440; 1.1797]	0.9159	[0.7754; 1.0818]	-1.03	0.3011
ESCT:IMT	0	0	.	.	0.9039	[0.6126; 1.3339]	.	.	.	.
ESCT:OISD	0	0	.	.	0.8928	[0.6723; 1.1855]	.	.	.	.
ESCT:OISD+E	0	0	.	.	0.8182	[0.6041; 1.1084]	.	.	.	.
ESCT:SCT	0	0	.	.	0.9228	[0.6927; 1.2292]	.	.	.	.
ESCT:SISD	0	0	.	.	0.8757	[0.6021; 1.2736]	.	.	.	.
ESCT:SMT	0	0	.	.	0.9122	[0.6838; 1.2168]	.	.	.	.
IMT:OISD	1	0.77	0.9697	[0.7144; 1.3162]	1.0492	[0.6023; 1.8277]	0.9242	[0.4905; 1.7416]	-0.24	0.8074
IMT:OISD+E	0	0	.	.	0.9052	[0.6806; 1.2039]	.	.	.	.
IMT:SCT	1	0.93	1.0303	[0.7817; 1.3579]	0.9069	[0.3378; 2.4352]	1.1360	[0.4074; 3.1681]	0.24	0.8074
IMT:SISD	0	0	.	.	0.9687	[0.6670; 1.4070]	.	.	.	.
IMT:SMT	0	0	.	.	1.0091	[0.7689; 1.3243]	.	.	.	.
OISD+E:OISD	2	0.38	0.9091	[0.7586; 1.0894]	1.2194	[1.0587; 1.4045]	0.7455	[0.5925; 0.9379]	-2.51	0.0122
SCT:OISD	8	0.78	0.9655	[0.9091; 1.0254]	0.9746	[0.8689; 1.0933]	0.9907	[0.8701; 1.1278]	-0.14	0.8871
SISD:OISD	0	0	.	.	1.0195	[0.7849; 1.3242]	.	.	.	.
SMT:OISD	5	0.67	0.9872	[0.9141; 1.0660]	0.9616	[0.8612; 1.0738]	1.0266	[0.8974; 1.1743]	0.38	0.7025
OISD+E:SCT	4	0.82	1.2088	[1.0757; 1.3583]	0.8184	[0.6369; 1.0515]	1.4771	[1.1202; 1.9475]	2.76	0.0057
OISD+E:SISD	0	0	.	.	1.0702	[0.8065; 1.4201]	.	.	.	.
OISD+E:SMT	0	0	.	.	1.1148	[0.9882; 1.2575]	.	.	.	.
SCT:SISD	0	0	.	.	0.9490	[0.7282; 1.2368]	.	.	.	.
SCT:SMT	6	0.69	0.9959	[0.9212; 1.0768]	0.9719	[0.8645; 1.0927]	1.0247	[0.8902; 1.1796]	0.34	0.7339
SISD:SMT	0	0	.	.	1.0416	[0.7981; 1.3596]	.	.	.	.

Legend:

© 2020 Wade RG et al. *JAMA Network Open.*

comparison - Treatment comparison

k - Number of studies providing direct evidence

prop - Direct evidence proportion

nma - Estimated treatment effect (RR) in network meta-analysis

direct - Estimated treatment effect (RR) derived from direct evidence

indir - Estimated treatment effect (RR) derived from indirect evidence

RoR - Ratio of Ratios (direct versus indirect)

z - z-value of test for disagreement (direct versus indirect)

p-value - p-value of test for disagreement (direct versus indirect)

© 2020 Wade RG et al. *JAMA Network Open.*

eTable 4. Direct and Indirect Estimates From the Random-Effects NMA of Complications

Comparisons of the direct and indirect estimates from the random-effects NMA of complications using the back-calculation method. Abbreviations as per Figure 1.

comparison	k	prop	direct	95%-CI	indir.	95%-CI	RoR	95%-CI	z	p-value
EISD:ESCT	1	1.00	0.9455	[0.1382; 6.4680]	.	.	.	.	.	.
EISD:IMT	0	0	.	.	4.6070	[0.2118; 100.1943]	.	.	.	.
EISD:OISD	3	1.00	2.2469	[0.8958; 5.6360]	.	.	.	.	.	.
EISD:OISD+E	0	0	.	.	8.1011	[0.7680; 85.4586]	.	.	.	.
EISD:SCT	0	0	.	.	1.3944	[0.4686; 4.1495]	.	.	.	.
EISD:SISD	1	1.00	3.2927	[0.1419; 76.3912]	.	.	.	.	.	.
EISD:SMT	0	0	.	.	1.1412	[0.3402; 3.8289]	.	.	.	.
ESCT:IMT	0	0	.	.	4.8727	[0.1291; 183.8770]	.	.	.	.
ESCT:OISD	0	0	.	.	2.3765	[0.2820; 20.0290]	.	.	.	.
ESCT:OISD+E	0	0	.	.	8.5685	[0.4094; 179.3332]	.	.	.	.
ESCT:SCT	0	0	.	.	1.4749	[0.1617; 13.4532]	.	.	.	.
ESCT:SISD	0	0	.	.	3.4826	[0.0874; 138.8471]	.	.	.	.
ESCT:SMT	0	0	.	.	1.2071	[0.1244; 11.7098]	.	.	.	.
IMT:OISD	0	0	.	.	0.4877	[0.0258; 9.2167]	.	.	.	.
IMT:OISD+E	0	0	.	.	1.7585	[0.0483; 63.9965]	.	.	.	.
IMT:SCT	1	0.87	0.3978	[0.0174; 9.0881]	0.0452	[0.0000; 173.2043]	8.8010	[0.0013; 59822.8395]	0.48	0.6291
IMT:SISD	0	0	.	.	0.7147	[0.0088; 58.2766]	.	.	.	.
IMT:SMT	1	0.96	0.2129	[0.0111; 4.0928]	7.0840	[0.0000; 7781670.3571]	0.0301	[0.0000; 45042.3744]	-0.48	0.6291
OISD+E:OISD	0	0	.	.	0.2774	[0.0317; 2.4271]	.	.	.	.
SCT:OISD	4	0.86	1.6796	[0.8938; 3.1563]	1.2408	[0.2547; 6.0447]	1.3536	[0.2462; 7.4423]	0.35	0.7277
SISD:OISD	0	0	.	.	0.6824	[0.0258; 18.0608]	.	.	.	.
SMT:OISD	4	0.67	1.6930	[0.6486; 4.4191]	2.6863	[0.6782; 10.6407]	0.6302	[0.1177; 3.3745]	-0.54	0.5897
OISD+E:SCT	2	1.00	0.1721	[0.0213; 1.3895]	.	.	.	.	.	.
OISD+E:SISD	0	0	.	.	0.4064	[0.0080; 20.6693]	.	.	.	.
OISD+E:SMT	0	0	.	.	0.1409	[0.0149; 1.3321]	.	.	.	.
SCT:SISD	0	0	.	.	2.3613	[0.0847; 65.8326]	.	.	.	.
SCT:SMT	5	0.56	0.7930	[0.2623; 2.3973]	0.8520	[0.2444; 2.9695]	0.9307	[0.1755; 4.9352]	-0.08	0.9328
SISD:SMT	0	0	.	.	0.3466	[0.0119; 10.0698]	.	.	.	.

Legend:

k - Number of studies providing direct evidence

© 2020 Wade RG et al. *JAMA Network Open.*

direct - Estimated treatment effect (OR) derived from direct evidence

indirect - Estimated treatment effect (OR) derived from indirect evidence

RoR - Ratio of Ratios (direct versus indirect)

z - z-value of test for disagreement (direct versus indirect)

p-value - p-value of test for disagreement (direct versus indirect)

© 2020 Wade RG et al. *JAMA Network Open.*

eTable 5. League Table of Pairwise Comparisons for Complications (Fixed-Effects Mantel-Haenszel)

League table of pairwise comparisons in the sensitivity network meta-analysis for complications using the fixed-effects Mantel-Haenszel method. Treatments are ordered by the rank chance (P score) of causing a complication with the least hazardous operation in the top left. Estimates are risk ratios (95% CIs). Large differences between treatments are highlighted in yellow.

Open in-situ decompression with epicondylectomy (P score = 0.1050)	1.36 [0.03; 70.20]	0.56 [0.29; 1.08]	0.63 [0.26; 1.53]	.	2.55 [1.02; 6.35]
0.51 [0.22; 1.15]	Open in-situ decompression (P score = 0.2165)	0.29 [0.06; 1.43]	.	.	.
0.57 [0.31; 1.07]	0.29 [0.06; 1.43]	Subcutaneous transposition (P score = 0.5793)	0.86 [0.27; 2.75]	.	.
1.94 [0.36; 10.39]	0.26 [0.04; 1.57]	0.89 [0.37; 2.12]	Submuscular transposition (P score = 0.6447)	.	.
2.70 [0.30; 24.28]	5.24 [0.33; 83.10]	1.54 [0.16; 15.14]	1.37 [0.13; 14.25]	Endoscopic subcutaneous transposition (P score = 0.6926)	0.94 [0.13; 6.95]
2.55 [1.02; 6.35]	4.94 [0.73; 33.38]	1.46 [0.48; 4.40]	1.29 [0.38; 4.40]	0.94 [0.13; 6.95]	Endoscopic in-situ decompression (P score = 0.7617)

© 2020 Wade RG et al. *JAMA Network Open.*

eTable 6. Direct and Indirect Estimates From the Fixed-Effects Mantel-Haenszel NMA of Complications

Comparisons of the direct and indirect estimates from the sensitivity fixed-effects Mantel-Haenszel NMA of complications using the SIDDE method.

Abbreviations as per Figure 1.

comparison	k	direct	95%-CI	indir.	95%-CI.1	RoR	95%-CI.2	z	p-value
EISD:ESCT	1	0.9434	[0.1280; 6.9547]	.	.	.	.	.	.
EISD:OISD	3	2.9784	[1.1136; 7.9659]	.	.	.	.	.	.
EISD:SCT	0	.	.	.	.	.	.	.	.
EISD:SMT	0	.	.	.	.	.	.	.	.
ESCT:OISD	0	.	.	.	.	.	.	.	.
ESCT:SCT	0	.	.	.	.	.	.	.	.
ESCT:SMT	0	.	.	.	.	.	.	.	.
SCT:OISD	4	1.8311	[0.9346; 3.5876]	1.8785	[0.2854; 12.3665]	0.9747	[0.1318; 7.2090]	-0.03	0.9800
SMT:OISD	4	1.7833	[0.7014; 4.5339]	3.3803	[0.6762; 16.8982]	0.5276	[0.0821; 3.3897]	-0.67	0.5005
SCT:SMT	5	0.7549	[0.2143; 2.6601]	0.6164	[0.1495; 2.5423]	1.2247	[0.1840; 8.1535]	0.21	0.8340

Legend:

k - Number of studies providing direct evidence

direct - Estimated treatment effect (OR) derived from direct evidence

indirect - Estimated treatment effect (OR) derived from indirect evidence

RoR - Ratio of Ratios (direct versus indirect)

z - z-value of test for disagreement (direct versus indirect)

p-value - p-value of test for disagreement (direct versus indirect)

© 2020 Wade RG et al. *JAMA Network Open.*

eTable 7. Comparisons of the Direct and Indirect Estimates From the Fixed-Effects NMA of Reoperation

Comparisons of the direct and indirect estimates from the fixed-effects NMA of reoperation using the SIDDE method. Abbreviations as per Figure 1.

comparison	k	nma	direct	indir.	RoR	z	p-value
EISD:ESCT	1	1.9245	1.9245	.	.	.	.
EISD:OISD	3	1.5895	1.5895	.	.	.	.
EISD:SCT	0	0.8406	.	.	.	.	.
EISD:SMT	0	0.3128	.	.	.	.	.
ESCT:OISD	0	0.8259	.	.	.	.	.
ESCT:SCT	0	0.4368	.	.	.	.	.
ESCT:SMT	0	0.1625	.	.	.	.	.
SCT:OISD	5	1.8908	1.8059	3.0711	0.5880	-0.30	0.7605
SMT:OISD	4	5.0818	4.9932	2.1700	2.3010	0.51	0.6119
SCT:SMT	2	0.3721	0.3088	0.5501	0.5613	-0.45	0.6534

Legend:

k - Number of studies providing direct evidence

direct - Estimated treatment effect (OR) derived from direct evidence

indirect - Estimated treatment effect (OR) derived from indirect evidence

RoR - Ratio of Ratios (direct versus indirect)

z - z-value of test for disagreement (direct versus indirect)

p-value - p-value of test for disagreement (direct versus indirect)

© 2020 Wade RG et al. *JAMA Network Open.*

eTable 8. Direct and Indirect Estimates From the Random-Effects NMA of Recurrence

Comparisons of the direct and indirect estimates from the random-effects NMA of recurrence using the back-calculation method. Abbreviations as per Figure 1.

comparison	k	prop	direct	95%-CI	indir.	95%-CI	RoR	95%-CI	z	p-value
EISD:ESCT	1	1.00	0.9455	[0.0374; 23.9276]	.	.	.	.	.	.
EISD:IMT	0	0	.	.	2.0889	[0.0423; 103.0387]	.	.	.	.
EISD:OISD	2	1.00	1.0536	[0.1683; 6.5938]	.	.	.	.	.	.
EISD:OISD+E	0	0	.	.	5.7075	[0.1302; 250.1072]	.	.	.	.
EISD:SCT	0	0	.	.	1.2006	[0.1414; 10.1944]	.	.	.	.
EISD:SFT	0	0	.	.	1.9025	[0.0109; 331.3389]	.	.	.	.
EISD:SMT	0	0	.	.	0.5143	[0.0534; 4.9538]	.	.	.	.
ESCT:IMT	0	0	.	.	2.2094	[0.0140; 349.3738]	.	.	.	.
ESCT:OISD	0	0	.	.	1.1144	[0.0271; 45.7682]	.	.	.	.
ESCT:OISD+E	0	0	.	.	6.0367	[0.0418; 871.9507]	.	.	.	.
ESCT:SCT	0	0	.	.	1.2698	[0.0264; 61.1843]	.	.	.	.
ESCT:SFT	0	0	.	.	2.0122	[0.0046; 886.6050]	.	.	.	.
ESCT:SMT	0	0	.	.	0.5440	[0.0105; 28.1392]	.	.	.	.
IMT:OISD	0	0	.	.	0.5044	[0.0162; 15.7330]	.	.	.	.
IMT:OISD+E	0	0	.	.	2.7323	[0.0251; 297.1464]	.	.	.	.
IMT:SCT	1	0.88	0.3978	[0.0113; 14.0207]	9.5558	[0.0005; 180659.7621]	0.0416	[0.0000; 1469.8200]	-0.59	0.5519
IMT:SFT	0	0	.	.	0.9108	[0.0026; 318.9924]	.	.	.	.
IMT:SMT	1	0.89	0.3548	[0.0101; 12.4608]	0.0146	[0.0000; 290.2269]	24.3775	[0.0007; 903680.1629]	0.59	0.5519
OISD+E:OISD	1	0.92	0.1484	[0.0047; 4.6905]	2.0038	[0.0000; 181229.9614]	0.0741	[0.0000; 11165.1980]	-0.43	0.6687
SCT:OISD	5	0.90	1.0306	[0.3230; 3.2884]	0.2046	[0.0062; 6.7185]	5.0367	[0.1271; 199.5179]	0.86	0.3891
SFT:OISD	0	0	.	.	0.5538	[0.0045; 68.8634]	.	.	.	.
SMT:OISD	3	0.68	2.0098	[0.4015; 10.0605]	2.1337	[0.2026; 22.4745]	0.9419	[0.0543; 16.3280]	-0.04	0.9672
OISD+E:SCT	1	0.86	0.2832	[0.0077; 10.3701]	0.0354	[0.0000; 238.7031]	7.9957	[0.0006; 109289.9567]	0.43	0.6687
OISD+E:SFT	1	1.00	0.3333	[0.0099; 11.1749]	.	.	.	.	.	.
OISD+E:SMT	0	0	.	.	0.0901	[0.0028; 2.9509]	.	.	.	.
SCT:SFT	0	0	.	.	1.5847	[0.0125; 200.8648]	.	.	.	.
SCT:SMT	3	0.69	0.2903	[0.0594; 1.4185]	1.0372	[0.0949; 11.3330]	0.2799	[0.0159; 4.9342]	-0.87	0.3844
SFT:SMT	0	0	.	.	0.2703	[0.0019; 38.1861]	.	.	.	.

Legend:

k - Number of studies providing direct evidence

direct - Estimated treatment effect (OR) derived from direct evidence

© 2020 Wade RG et al. *JAMA Network Open.*

indirect - Estimated treatment effect (OR) derived from indirect evidence

RoR - Ratio of Ratios (direct versus indirect)

z - z-value of test for disagreement (direct versus indirect)

p-value - p-value of test for disagreement (direct versus indirect)

© 2020 Wade RG et al. *JAMA Network Open.*

eTable 9. League Table of Pairwise Comparisons for Recurrence (Fixed-Effects Mantel-Haenszel)

League table of pairwise comparisons for the risk of recurrence network meta-analysis for complications using the fixed-effects Mantel-Haenszel method. Treatments are ordered by the rank chance (P-score) of causing a complication with the lowest risk operation in the top left. Estimates are risk ratios (95% CIs). Large differences between treatments are highlighted in yellow.

Endoscopic in-situ decompression (P score = 0.3673)	0.95 [0.26; 3.50]	.	0.94 [0.06; 15.50]	.
0.95 [0.26; 3.50]	Open in-situ decompression (P score = 0.3783)	0.91 [0.42; 1.94]	.	0.28 [0.10; 0.77]
0.93 [0.21; 4.21]	0.98 [0.46; 2.09]	Subcutaneous transposition (P score = 0.3931)	0.15 [0.04; 0.65]	.
0.94 [0.06; 15.50]	1.01 [0.05; 22.10]	0.99 [0.04; 23.72]	Endoscopic subcutaneous transposition (P score = 0.4284)	.
0.23 [0.04; 1.32]	0.24 [0.07; 0.78]	0.24 [0.07; 0.83]	0.24 [0.01; 6.54]	Submuscular transposition (P score = 0.9328)

© 2020 Wade RG et al. *JAMA Network Open.*

eTable 10. Direct and Indirect Estimates From the Fixed-Effects Mantel-Haenszel NMA of Recurrence

Comparisons of the direct and indirect estimates from the fixed-effects Mantel-Haenszel NMA of recurrence using the SIDDE method. Abbreviations as per Figure 1.

comparison	k	nma	direct	indir.	RoR	z	p-value
EISD:ESCT	1	0.9444	0.9444	.	.	.	.
EISD:OISD	2	0.9536	0.9536	.	.	.	.
EISD:SCT	0	0.9335	.	.	.	.	.
EISD:SMT	0	0.2256	.	.	.	.	.
ESCT:OISD	0	1.0097	.	.	.	.	.
ESCT:SCT	0	0.9884	.	.	.	.	.
ESCT:SMT	0	0.2389	.	.	.	.	.
SCT:OISD	5	1.0215	1.1025	0.1752	6.2924	0.96	0.3367
SMT:OISD	3	4.2259	3.5453	1.2577	2.8188	0.65	0.5170
SCT:SMT	3	0.2417	0.1522	5.5884	0.0272	-2.48	0.0132

Legend:

k - Number of studies providing direct evidence

direct - Estimated treatment effect (OR) derived from direct evidence

indirect - Estimated treatment effect (OR) derived from indirect evidence

RoR - Ratio of Ratios (direct versus indirect)

z - z-value of test for disagreement (direct versus indirect)

p-value - p-value of test for disagreement (direct versus indirect)

© 2020 Wade RG et al. *JAMA Network Open.*

eTable 11. CINEMA Assessments for the Primary Outcome

The CINEMA assessment of the confidence in the results for the primary outcome. Abbreviations as per Figure 1.

Comparison	Number of studies	Within-study bias	Reporting bias	Indirectness	Imprecision	Heterogeneity	Incoherence	Confidence rating
Mixed Evidence								
EISD:ESCT	1	Major concerns	Suspected	--	Major concerns	No concerns	No concerns	Moderate
EISD:OISD	6	Major concerns	Suspected	--	Major concerns	No concerns	No concerns	Moderate
EISD:SCT	1	Major concerns	Suspected	--	Major concerns	No concerns	No concerns	Moderate
EISD:SISD	1	Major concerns	Suspected	--	Major concerns	No concerns	No concerns	Moderate
EISD:SMT	1	Major concerns	Suspected	--	Major concerns	No concerns	No concerns	Moderate
IMT:OISD	1	Major concerns	Suspected	--	Major concerns	No concerns	No concerns	Moderate
IMT:SCT	1	Major concerns	Suspected	--	Major concerns	No concerns	No concerns	Moderate
OISD:OISD+E	2	Major concerns	Suspected	--	Major concerns	No concerns	Major concerns	Very low
OISD:SCT	8	Major concerns	Suspected	--	Major concerns	No concerns	No concerns	Moderate
OISD:SMT	5	Major concerns	Suspected	--	Major concerns	No concerns	No concerns	Moderate
OISD+E:SCT	4	Major concerns	Suspected	--	No concerns	Major concerns	Major concerns	Very low
SCT:SMT	6	Major concerns	Suspected	--	Major concerns	No concerns	No concerns	Moderate
Indirect Evidence								
EISD:IMT	0	Major concerns	Suspected	--	Major concerns	No concerns	No concerns	Low
EISD:OISD+E	0	Major concerns	Suspected	--	Major concerns	No concerns	No concerns	Low

© 2020 Wade RG et al. *JAMA Network Open.*

Comparison	Number of studies	Within-study bias	Reporting bias	Indirectness	Imprecision	Heterogeneity	Incoherence	Confidence rating
ESCT:IMT	0	Major concerns	Suspected	--	Major concerns	No concerns	No concerns	Low
ESCT:OISD	0	Major concerns	Suspected	--	Major concerns	No concerns	No concerns	Low
ESCT:OISD+E	0	Major concerns	Suspected	--	Major concerns	No concerns	No concerns	Low
ESCT:SCT	0	Major concerns	Suspected	--	Major concerns	No concerns	No concerns	Low
ESCT:SISD	0	Major concerns	Suspected	--	Major concerns	No concerns	No concerns	Low
ESCT:SMT	0	Major concerns	Suspected	--	Major concerns	No concerns	No concerns	Low
IMT:OISD+E	0	Major concerns	Suspected	--	Major concerns	No concerns	No concerns	Low
IMT:SISD	0	Major concerns	Suspected	--	Major concerns	No concerns	No concerns	Low
IMT:SMT	0	Major concerns	Suspected	--	Major concerns	No concerns	No concerns	Low
OISD:SISD	0	Major concerns	Suspected	--	Major concerns	No concerns	No concerns	Low
OISD+E:SISD	0	Major concerns	Suspected	--	Major concerns	No concerns	No concerns	Low
OISD+E:SMT	0	Major concerns	Suspected	--	No concerns	No concerns	No concerns	Moderate
SCT:SISD	0	Major concerns	Suspected	--	Major concerns	No concerns	No concerns	Low
SISD:SMT	0	Major concerns	Suspected	--	Major concerns	No concerns	No concerns	Low

© 2020 Wade RG et al. *JAMA Network Open.*

eAppendix. Search Results

PubMed

1. (release).ti,ab (623,041)
2. (decompression).ti,ab (43,555)
3. (endoscopic).ti,ab (416,274)
4. (epicondyle*).ti,ab (1829)
5. (transposition).ti,ab (24,503)
6. (ulnar nerve).ti,ab (12,126)
7. (ulnar neuritis).ti,ab (2524)
8. (cubital tunnel).ti,ab (1021)
9. (ulnar neuropathy).ti,ab (3500)
10. (12 OR 13 OR 5 OR 7 OR 11) (1,096,837)
11. (8 OR 9 OR 6 OR 10) (12,625)
12. (14 AND 15) (1828)

Embase

1. (release).ti,ab (652,499)
2. (decompression).ti,ab (43,689)
3. (endoscopic).ti,ab (211,851)

© 2020 Wade RG et al. *JAMA Network Open.*

4. (epicondyle*).ti,ab (2227)
5. (transposition).ti,ab (24,783)
6. (ulnar nerve).ti,ab (7311)
7. (ulnar neuritis).ti,ab (109)
8. (cubital tunnel).ti,ab (1016)
9. (ulnar neuropathy).ti,ab (894)
10. (12 OR 13 OR 5 OR 7 OR 11) (928,006)
11. (8 OR 9 OR 6 OR 10) (8178)
12. (14 AND 15) (1508)

CENTRAL

"Cubital tunnel"

77 trials

2 reviews

© 2020 Wade RG et al. *JAMA Network Open.*
